# Improvement in energy performance from the construction of inlet guide vane and diffuser vane geometries in an axial-flow pump

**DOI:** 10.1038/s41598-024-51220-6

**Published:** 2024-01-03

**Authors:** Duc-Anh Nguyen, Cong-Truong Dinh, Jin-Hyuk Kim

**Affiliations:** 1 Convergence Manufacturing System Engineering (Green Process and Energy System Engineering), University of Science & Technology, Daejeon, 34113 South Korea; 2 Carbon Neutral Technology R&D Department, Korea Institute of Industrial Technology, Cheonan, 31056 South Korea; 3 School of Mechanical Engineering, Hanoi University of Science and Technology, Hanoi, 11615 Vietnam

**Keywords:** Mechanical engineering, Engineering

## Abstract

Advanced inlet guide vane (IGV) and diffuser vane (DV) geometries were constructed in an effort to increase the energy performance of an axial-flow pump at the best efficiency point (BEP). DV setting angles were also investigated to increase energy performance at the off-design points. By integrating the advantages of an adjustable IGV, combinations of adjustable IGV and DV geometries were constructed and thoroughly analyzed by way of energy loss. The asymmetrical geometry of the IGV, upgraded through the use of a hydrofoil profile, resulted in higher hydraulic performance compared to that of the reference model. The efficiency and total head at the BEP increased significantly with the implementation of the new DV, by 1.456% and 5.756% over those of the reference model, respectively. Using the new DV reduced the unsteady turbulent flow behind the trailing edge of the DV under all flow rate conditions, resulting in a reduction in vibration and noise. The positive setting angles of the DV increased the energy performance in the high-flow-rate region, whereas the negative DV setting angles produced a good performance in the low-flow-rate region. Combining an adjustable IGV with an adjustable DV model resulted in a significant increase in the total head, with more optimal energy performance provided by the positive IGV setting angles. At the BEP and under high-flow-rate conditions, the low-velocity zone is closely related to high entropy generation. Furthermore, these high-entropy generation regions follow the trajectory of the low-velocity zones. However, the low-velocity zone is not strongly associated with the high-entropy generation region when operating under low-flow-rate conditions.

## Introduction

An axial-flow pump, a type of fluid pumping device, utilizes an impeller to produce a fluid flow parallel to the rotational shaft of the pump. Its main applications are in irrigation, agriculture, water supply, and drainage plants, where the large volume of fluid flow is a key factor. An axial-flow pump can operate under low-pressure conditions with a high-flow-rate, which makes it suitable for transferring large fluid mass at low viscosity. Additionally, the axial-flow pump has a compact design, resulting in easier installation and maintenance. However, the axial-flow pump cannot easily produce a high pressure head, as its impeller is designed to operate well at high flow volume. According to Capurso et al.^1^, the power consumption of centrifugal pumps in Europe is 120 TWh/day corresponding to an average carbon production of 475 gCO_2_/kWh, and with a 1% improvement in energy performance, it results in a reduction of the greenhouse effect at least $-$ 570 tCO_2_/day. In fact, the amount of power required to operate an axial-flow pump is much larger than that of a centrifugal pump because of its large size. Therefore, optimizing the axial-flow pump to increase efficiency and the pressure head is necessary for efficient pump operation, energy saving and environmental protection.

The axial-flow pump often consists of an inlet guide vane (IGV), impeller, and diffuser vane (DV). The operation of the pump is directly affected by the geometry of these main components. The IGV is a device widely used in turbomachinery and in axial-flow pumps; it can help reduce the turbulence in the flow before it enters the impeller, creating a soft fluid flow that functions under low-flow-rate conditions^2^. Additionally, by rotating the IGV setting angle, the IGV can increase or decrease the flow rate and control the direction of the fluid flow in front of the impeller^3–7^. In addition to the IGV, some new design methods have been attempted in an effort to control the flow in front of the impeller; such methods have included the use of grooves^8,9^ and vortex generators^10,11^. However, the common factor in all these studies is that they do not focus on the shape of the IGV, as there has been no study on the influence of the IGV shape until now. Nowadays, the IGV shape is typically symmetric, with the primary function of directing the flow before it enters the impeller. According to Yang et al.^7^, the research pump model includes eleven conical-tip symmetrical IGVs. The Kim et al.^5^ and Nguyen et al.^6^ studies include an IGV taking the form of a simple blade rounded at the leading edge (LE) and trailing edge (TE) with constant thickness. Furthermore, in the studies of Feng et al.^3^ and Guo et al.^4^, the shape of the IGV is rather rudimentary, with a rectangular profile. Grooves and vortex generators have also been adopted outside of the IGV, with rough shapes with rectangular profiles. This produces a large drag at the IGV, causing significant hydraulic loss and greatly affecting the energy efficiency of the axial-flow pump. Consequently, an IGV with a hydrofoil profile is proposed in this study with the aim of increasing the efficiency and pressure head of the axial-flow pump in comparison with those of the conventional profile.

The IGV in the axial-flow pump is not normally designed with a hydrofoil profile, as the IGV is a stationary blade and lift or propulsion in an IGV is not frequently required. However, some modern designs have used a hydrofoil profile in the IGV geometry to optimize fluid flow, boost energy performance, and decrease vibration. Hydrofoil profiles are used in underwater blades, which produce lift when the fluid flow passes through them. This lift allows the hydrofoil to decrease drag, which makes it glide through the water more quickly and effectively. There are many types of hydrofoil used in practice, such as the surface-piercing hydrofoil, fully submerged hydrofoil, T-foil hydrofoil, and flapping hydrofoil. Consequently, hydrofoil is an advanced technology that may be employed in many different applications to increase energy performance and reduce energy loss. The NACA foil, also referred to as the NACA airfoil, was created for the same purpose as the hydrofoil. Airfoil profiles were initially developed for aviation wings; however, their good streamline and energy performance features also make them suitable for other applications, such as hydrofoil design. Although there are other options for hydrofoil design, airfoil profiles provide a well-established and verified starting point for producing streamlined geometries that are optimized for specific applications and operating conditions.

The DV can increase the efficiency and pressure head of the axial-flow pump by converting the fluid kinetic energy into pressure energy, enabling it to pump large volumes of fluid with higher pressure, which is essential for many applications. The design of the DV is critical to the efficiency, total head, power consumption, entropy generation, and other parameters that can be generally called energy performance. The number, shape, and spacing of the vanes in the DV can be optimized to maximize pressure recovery and minimize hydraulic loss and energy dissipation owing to turbulence. According to Kim et al.^12^, the impeller and DV were optimized to simultaneously increase both the total efficiency and pressure head of the axial-flow pump through a hybrid multi-objective evolutionary algorithm, coupled with a response surface approximation model. The results showed that the total efficiency and pressure head of the optimum model increased by 9.03% and 4.61%, respectively, in comparison with that of the reference model. According to Nguyen et al.^13^, optimization technology was also applied to the impeller and DV to improve the performance of the axial-flow pump. Consequently, the efficiency and pressure head of the optimum model increased by 3.097% and 10.205%, respectively, compared to those of the reference model. In addition, the performance increased due to the reduction in turbulence in the DV passage. Along with the axial-flow pump, the mixed-flow pump underwent DV optimization to improve hydraulic performance^14–16^. In summary, the results of these studies showed that the efficiency of the axial-flow pump is highly dependent on the design of the DV. In theory, the DV passage is characterized by a gradually larger cross-sectional area in the flow path, which reduces the fluid velocity and increases the pressure^17^. This method has been applied in many different axial-flow pumps^2,18–21^. In fact, even after optimization, this type of profile is still used^12,13^. However, there are many other studies that employ a straight hub and shroud profiles inside the DV passage, instead of a gradually increasing area profile^22–25^. Changing the cross-sectional area of the DV has a great influence on the energy performance of the axial-flow pump. Consequently, the hub profile inside the DV passage is investigated and analyzed in detail in this study.

The IGV and DV are typically designed to provide the highest energy performance at the best efficiency point (BEP)^12,13^. Consequently, the energy performance at the off-design points is often severely reduced. To improve performance in these regions, adjustable IGV and DV are proposed. According to Shi et al.^26^ and Qian et al.^27^, the energy performance of the axial-flow pump was increased at the off-design points with the rotation of the DV at the center of the chord length. Similar studies have also been performed to produce an improvement in energy performance via a change in the inlet angle of the DV^28,29^. For the IGV, the influence of an adjustable IGV was also examined, whereby the adjustable IGV was shown to have a positive effect on the energy performance of the axial-flow pump^6,7^. The influences of the rotation angles of the IGV and DV are very significant to the energy performance of the axial-flow pump at the off-design points. However, there is no study showing the effect of the simultaneous rotation of the DV and IGV. Therefore, combinations of the adjustable IGV and adjustable DV were investigated and thoroughly analyzed in this study.

No previous studies have shown the influences of the IGV and DV hub profiles on the energy performance of the axial-flow pump. To increase the efficiency and total pressure head, the profiles of the IGV and DV hub are modified and analyzed in detail by comparison with the reference shape. In this modification, the IGV is proposed to use a NACA foil profile, and the hub in the DV passage is considered to adopt a straight profile. In addition, to increase the energy performance of the axial-flow pump at the off-design points, an adjustable DV is proposed and thoroughly investigated. Finally, combinations of simultaneously rotating IGV and DV are performed and analyzed in detail.

### Hypotheses and theoretical analysis

#### Effect of the IGV profile

**Figure 1:**
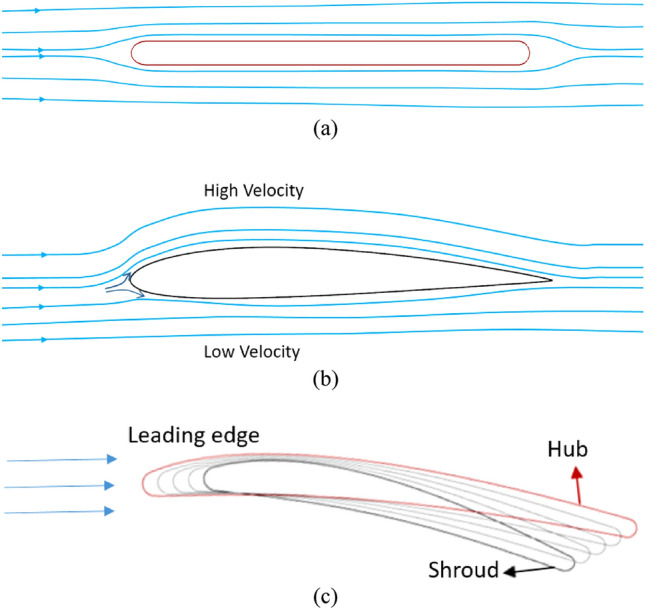
IGV profiles. (**a**) Symmetrical profile, (**b**) NACA foil profile, and (**c**) New IGV design.

The Figure 1 presents the two types of IGV geometries that are investigated in this study with a normal profile and NACA foil profile. NACA airfoils^30,31^ are designed to produce high lift with minimal drag and smooth flow. Consequently, the IGV with NACA foil profile produces a high-energy flow with less loss owing to turbulence and flow separation, compared to that of the normal IGV shape, which can be important for reducing vibration and noise. In addition, the hydrofoil with NACA profile decreased drag and increased momentum and pressure in the flow through the blades. This is very useful in the case of pumps operating under low-flow-rate conditions, in which cavitation is likely to occur owing to an excessive inlet pressure drop. Furthermore, the hydrofoil with NACA profile may be able to control the fluid flow direction, which may be essential to ensuring that the fluid flow is guided efficiently through the IGV passage and does not result in any damage. Then, the flow, after passing through the IGV with the NACA foil profile, reaches the impeller with a good flow direction, leading to an enhancement in the energy performance.

#### Effect of the DV cross-sectional area

The DV passage in the axial-flow pump is frequently designed with a progressive enhancement in the cross-sectional area in the flow direction^17,32^. This facilitates the conversion of kinetic energy into pressure energy, which is subsequently applied to increase the pressure head of the fluid^17^. Therefore, the distribution of the cross-sectional area inside the DV passage is a crucial design parameter. A larger cross-sectional area in the DV passage results in a progressive decrease in the fluid velocity and kinetic energy, leading to higher pressure recovery and energy performance. Nevertheless, there are also practical restrictions on how large the cross-sectional area can be, depending on various factors such as the pump size and available DV space. In the case that the cross-sectional area of the DV is not optimized, separation may occur under the action of an adverse pressure gradient, if its area is increased too suddenly^17,33^. This is the key reason for the decrease in the energy performance and increase in entropy generation in the DV of the axial-flow pump.

#### Adjustable DV

**Figure 2:**
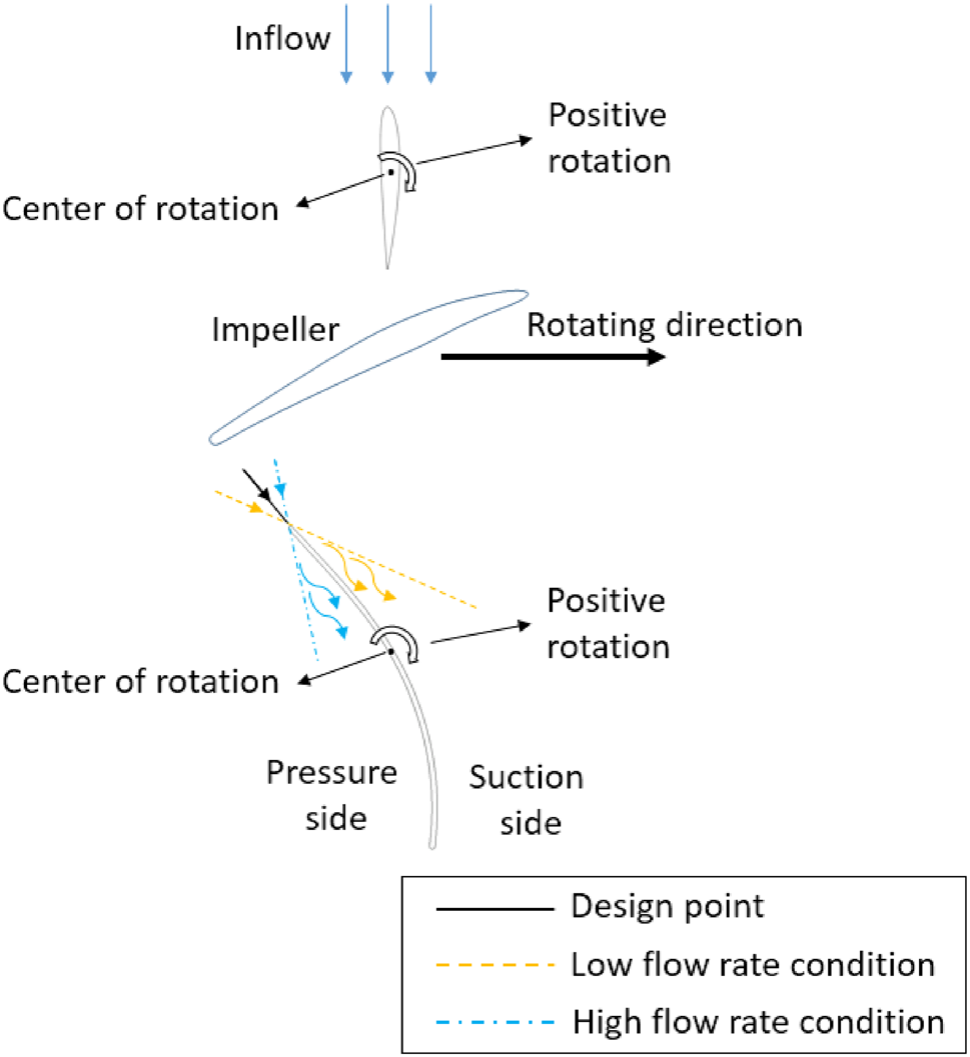
Two-dimensional illustration of the IGV and DV setting angles.

To enhance the energy performance of the axial-flow pump at the off-design points, varying the setting angle of the DV is an effective method. Figure 2 illustrates a two-dimensional drawing of the setting angle of the DV. The DV setting angle is considered as the reference angle as it is in the design mode. The position of the DV is referred to as a positive setting angle when it is rotated clockwise; otherwise, it is designated as a negative angle. The general principle of these changes is to match the flow and blade angle at the LE of the DV to create a smooth flow inside the passage of the DV. In the positive setting angle case, matching is improved by suppressing the separation flow at the pressure side (PS) of the DV, which is induced by a low flow angle. Consequently, the hydraulic losses are improved, and the energy performance is significantly increased. Likewise, the separation flow at the suction side (SS) of the DV under low-flow-rate conditions is also eliminated at negative DV setting angles. The turbulent flow in the DV passage is improved, resulting in increased energy performance.

#### Adjustable IGV and DV

Changing the setting angles of the IGV was analyzed in detail in a previous study^6^ and was demonstrated to be effective in improving the energy performance of the axial-flow pump. In the present study, simultaneous rotation of the IGV and DV is assumed to be highly effective, combining the benefits of both rotating methods. Both the efficiency and total pressure head of the axial-flow pump are thought to increase, owing to the changing of the IGV setting angle, which produces a flow with higher energy at the impeller outlet and eliminates turbulent flow in the DV passage.

## Methodology

### Experimental setup

**Figure 3:**
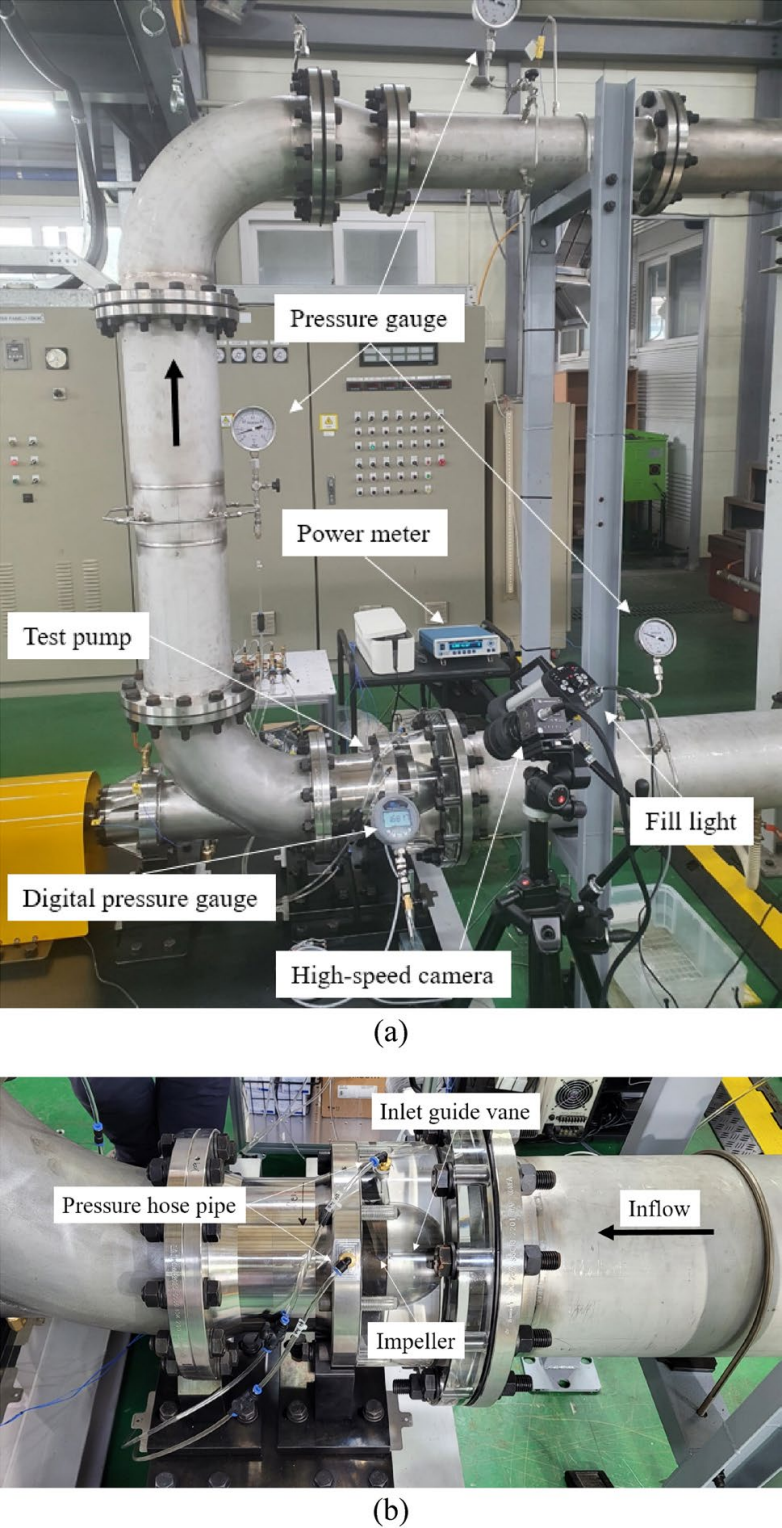
Axial-flow pump test. (**a**) Test system and (**b**) Test pump.

Prototypes in the axial-flow pump were manufactured and underwent experimentation at the Korea Institute of Machinery and Materials. Figure 3 presents the test system of the axial-flow pump. The experiment was conducted in a closed pipeline system, using transparent material at the IGV and impeller shrouds to facilitate supervision of the flow inside the axial-flow pump for instance cavitation phenomenon, flow direction, and separation flow, etc. The experiment consisted of an electric motor, pressure gauge, digital power meter, flow meter, control gate valve, high-speed camera, water tank, and fill light. To obtain a stable signal, a flow meter was installed at a distance from the pump test. To sustain a steady flow, both inlet and outlet pipelines were stretched far from the rotating impeller to prevent backflow influence. A motor shaft was coupled to a measurement system placed behind the experimental system to gauge the shaft power. Detailed descriptions of the test system and uncertainty values of the measurement devices were presented in a previous study^6^.

#### Numerical method

Computational fluid dynamics (CFD)^34^ is an application of mathematics that employs numerical schemes and algorithms to calculate and analyze fluid flow phenomena. Simulations in CFD consist of constructing a computational model, establishing boundary conditions, and solving the governing equations by numerical means. The simulation results are used to deeply analyze the flow phenomena described by the computational model and can be used to further optimize the system design. With the use of CFD simulations, engineers can predict the efficiency of a system, identify problems, and construct more optimal designs to obtain great performance and decrease energy consumption. In addition, the use of CFD simulations may decrease experimental and prototyping costs and increase the safety of products and systems by identifying the phenomena inside the fluid flow in a variety of situations. Most importantly, the results of numerical simulations are highly accurate, allowing engineers to analyze in detail the fluid flow behavior that may not be analyzable with experimental tests alone.

In this work, to describe the fluid-flow physics and hydraulic properties of the axial-flow pump, ANSYS CFX software is used. It is widely known as software that applies the laws of physics, such as the conservation of mass, energy, and momentum, to solve complex fluid dynamics problems, including those involving pumps. Numerical results are gained by applying the 3D Reynolds-averaged Navier-Stokes equations (RANS) for continuous incompressible flow. Here, the finite volume method is employed to discretize the RANS equations. The governing equations, consisting of the continuity equation (Eq. (1)) and momentum equation (Eq. (2)), may be described as follows:

1 $$\frac{\partial \rho }{{\partial t}} + \frac{{\partial \left( {\rho u_{i} } \right)}}{{\partial x_{i} }} = 0$$

2 $$\rho \left[ {\frac{{\partial \left( {u_{i} } \right)}}{\partial t} + \frac{{\partial \left( {u_{i} u_{j} } \right)}}{{\partial x_{j} }}} \right] = - \frac{\partial p}{{\partial x_{i} }} + \frac{\partial }{{\partial x_{j} }}\left[ {\mu \frac{{\partial u_{i} }}{{\partial x_{j} }} - \rho \overline{{u_{i}{\prime} u_{j}{\prime} }} } \right]$$

where, $\rho \overline{{u }_{i}{\prime}{u}_{j}{\prime}}$, $\rho$, $t$, ${u}_{i}$, $\mu$, and $p$ denote the Reynolds stress, fluid density, time, fluid velocity components, turbulent eddy viscosity, and local static pressure strength, respectively.

#### Turbulence model

In the present study, to follow the phenomena occurring near the wall, the shear stress transport (SST $k-\omega$) turbulence model, coupled with reattachment modification, is used. The flow separation point, which is located in the region near the wall where adverse pressure gradients are present, may be predicted rather well using the SST turbulence model created by Menter^35^. Nevertheless, the reattachment points are frequently forecasted too far downstream compared to what is observed experimentally, a well-known weakness in all turbulence models. By including the source term "${P}_{reattach}$" in the transport equation for the turbulence kinetic energy (TKE) (Eq. (3)), the SST turbulence model was modified, increasing the forecasting capability of the reattaching position^36^ without a negative impact on the accuracy of the SST turbulence model for attached and weakly separated boundary layers. The SST turbulence model coupled with reattachment modification may be described as follows:

3 $$\frac{{\partial \left( {\rho k_{i} } \right)}}{\partial t} + \frac{{\partial \left( {\rho \overline{{U_{j} }} k} \right)}}{{\partial x_{j} }} = P_{k} - \beta^{*} \rho k\omega + \frac{\partial }{{\partial x_{j} }}\left[ {\left( {\mu + \frac{{\mu_{t} }}{{\sigma_{k} }}} \right)\frac{\partial k}{{\partial x_{j} }}} \right] + P_{reattach}$$

4 $$\frac{{\partial \left( {\rho \omega } \right)}}{\partial t} + \frac{{\partial \left( {\rho u_{i} \omega } \right)}}{{\partial x_{j} }} = \frac{\gamma }{{\vartheta_{t} }}P_{k} - \beta \rho \omega^{2} + \frac{\partial }{{\partial x_{j} }}\left[ {\left( {\mu + \sigma_{\omega } \mu_{t} } \right)\frac{\partial \omega }{{\partial x_{j} }}} \right] + 2\left( {1 - F_{1} } \right)\frac{{\rho \sigma_{{\omega^{2} }} }}{\omega }\frac{\partial k}{{\partial x_{j} }}\frac{\partial \omega }{{\partial x_{j} }}$$

5 $$\mu_{t} = {\text{min}}\left( {\frac{\rho k}{\omega }, \;\frac{{\rho a_{1} k_{u} }}{{SF_{2} }}} \right)$$

6 $$P_{reattach} = P_{k} .min\left[ {4max\left( {0,\;\frac{{min\left( {S^{2} , {\Omega }^{2} } \right)}}{{0.09\omega^{2} }} - 1.6} \right), \;1.5} \right]$$

7 $$\emptyset = F_{1} \emptyset_{1} + \left( {1 - F_{1} } \right)\emptyset_{2}$$

where $k$, ${P}_{k}$, $\omega$, ${\mu }_{t}$, ${\vartheta }_{t}$, S, $\Omega$, and $\varnothing$ are the TKE, production term, turbulent frequency, molecular dynamic viscosity, turbulent kinematic viscosity, invariant measurement of strain rate, vorticity intensity, and a constant, respectively.

The SST turbulence model combines the advantages of the $k-\omega$ and $k- \varepsilon$ turbulence models with a blending function (F1) that produces a soft transition between them. The $k-\omega$ turbulence model uses a small grid size to capture the phenomena occurring near the wall surface, whereas the $k- \varepsilon$ turbulence model analyzes the turbulence in the main flow region, using a larger grid size. A wall function is employed in the computation to obtain a soft transition between the two regimes. The $\beta$, ${\sigma }_{k}$, and ${\sigma }_{\omega }$ parameters are determined through Eq. (7), applying the following constants^35^: ${\beta }_{1}=0.075$, ${\beta }_{2}=0.0828$, ${\sigma }_{k1}=0.85$, ${\sigma }_{k2}=1.0$, ${\sigma }_{\omega 1}=0.5$, ${\sigma }_{\omega 2}=0.856$, and ${\beta }^{*}=0.09$.

#### Entropy generation theory

Operating the axial-flow pump under off-design conditions causes much turbulence, leading to a large energy loss that cannot be irreversibly transformed into internal energy. Here, entropy generation theory can be effectively used to determine the energy dissipation caused by turbulence and viscous friction in fluid flow. In the turbulent flow, the local entropy generation rate^37^ can be described as having two sources: direct dissipation, which is induced by the time-averaged velocity, and turbulent dissipation, which is caused by the velocity oscillation. For the flow in the axial-flow pump and no heat exchange with the surroundings, the transport equation of the entropy generation rate is not entirely consistent with the commonly entropy transport equation^38,39^ and it can be expressed as follows:

8 $$\rho \frac{\partial s}{{\partial t}} + \rho u_{i} \frac{\partial s}{{\partial x_{j} }} = div\left( \frac{q}{T} \right) + \dot{S}_{D}^{\prime \prime \prime }$$

9 $$\dot{S}_{D}^{\prime \prime \prime } = \frac{{\mathop {Q_{t} }\limits }}{T} = m_{ji} \frac{{\partial u_{i} }}{{\partial x_{j} }}$$

10 $$m_{ji} = \mu \left( {\frac{{\partial u_{i} }}{{\partial x_{j} }} + \frac{{\partial u_{j} }}{{\partial x_{i} }}} \right) - \frac{{2\mu \delta_{ij} }}{3}\frac{{\partial u_{i} }}{{\partial x_{i} }}$$

where $s$, $q$, ${m}_{ji}$, ${\dot{S}}_{D}^{{\prime}{\prime}{\prime}}$, ${Q}_{t}$, $T$ and ${\delta }_{ij}$ are specific entropy, the heat flux, the viscous stress tensor, the local entropy generation rate, energy transfer rate, temperature, and the Kronecker delta symbol, respectively. In the numerical simulation with RANS, the velocity in turbulent flow can be decomposed into time-averaged ($\overline{u }$) and fluctuating components (${u}{\prime}$). Therefore, by applying the continuity equation for continuous incompressible flows, the entropy generation rate can be described as consisting of the following two components.

11 $$\dot{S}_{{\overline{D}}}^{\prime \prime \prime } = \frac{2\mu }{T} \left[ {\left( {\frac{{\partial \overline{u}_{1} }}{{\partial x_{1} }}} \right)^{2} + \left( {\frac{{\partial \overline{u}_{2} }}{{\partial x_{2} }}} \right)^{2} + \left( {\frac{{\partial \overline{u}_{3} }}{{\partial x_{3} }}} \right)^{2} } \right] + \frac{\mu }{T}\left[ {\left( {\frac{{\partial \overline{u}_{2} }}{{\partial x_{1} }} + \frac{{\partial \overline{u}_{1} }}{{\partial x_{2} }}} \right)^{2} + \left( {\frac{{\partial \overline{u}_{3} }}{{\partial x_{1} }} + \frac{{\partial \overline{u}_{1} }}{{\partial x_{3} }}} \right)^{2} + \left( {\frac{{\partial \overline{u}_{2} }}{{\partial x_{3} }} + \frac{{\partial \overline{u}_{3} }}{{\partial x_{2} }}} \right)^{2} } \right]$$

12 $$\dot{S}_{{D^{\prime}}}^{\prime \prime \prime } = \frac{{2\mu_{eff} }}{T}\left[ {\left( {\frac{{\partial u_{1}{\prime} }}{{\partial x_{1} }}} \right)^{2} + \left( {\frac{{\partial u_{2}{\prime} }}{{\partial x_{2} }}} \right)^{2} + \left( {\frac{{\partial u_{3}{\prime} }}{{\partial x_{3} }}} \right)^{2} } \right] + \frac{{\mu_{eff} }}{T}\left[ {\left( {\frac{{\partial u_{2}{\prime} }}{{\partial x_{1} }} + \frac{{\partial u_{1}{\prime} }}{{\partial x_{2} }}} \right)^{2} + \left( {\frac{{\partial u_{3}{\prime} }}{{\partial x_{1} }} + \frac{{\partial u_{1}{\prime} }}{{\partial x_{3} }}} \right)^{2} + \left( {\frac{{\partial u_{2}{\prime} }}{{\partial x_{3} }} + \frac{{\partial u_{3}{\prime} }}{{\partial x_{2} }}} \right)^{2} } \right]$$

where $\dot{S}_{{\overline{D} }}^{\prime \prime \prime }$ and ${\dot{S}}_{D}^{{\prime}{\prime}{\prime}}$ represent the entropy generation rate caused by direct dissipation (EPDD) and the rate caused by turbulent dissipation (EPTD), respectively. Parameter ${\mu }_{eff}$ denotes the effective dynamic viscosity of the fluid. The whole turbulent viscosity and dynamic viscosity make up the effective dynamic viscosity.

Because the numerical results are determined using the Reynolds-averaged approach, the velocity oscillation components are unavailable. To address this problem, turbulence models can be employed to calculate the EPTD^40,41^. In this study, owing to the use of the SST $k-\omega$ turbulence model, the EPTD may be expressed as follows:

13 $$\dot{S}_{{D^{\prime}}}^{\prime \prime \prime } = \beta^{*} \frac{\rho \omega k}{T}$$

Energy losses in the tip clearance region of the impeller are quite large under high rotating speed conditions because of the high velocity gradient. Therefore, Duan et al.^42^ additionally developed a wall function to determine the near-wall entropy generation rate (${\dot{S}}_{w}$), which is caused by the wall shear stress and is represented as follows:

14 $$\dot{S}_{w} = \mathop \smallint \limits_{A} \frac{{\tau_{w} .v_{w} }}{T}dA$$

where ${\tau }_{w}$, ${v}_{w}$, and $A$ represent the shear stress in the wall, near-wall velocity, and area, respectively.

In this study, the entropy generation occurring in the DV passage is mainly caused by the difference in the velocity gradient and viscosity dissipation. Therefore, the local entropy generation rate consists of EPDD and EPTD, which can be calculated by integrating over the entire volume of the DV region. It can be expressed as follows:

15 $$\dot{S}_{{\overline{D}}} = \int\limits_{V} {\dot{S}_{{\overline{D}}}^{\prime \prime \prime } dV}$$

16 $$\dot{S}_{D\prime } = \mathop \smallint \limits_{V} \dot{S}_{D\prime }^{\prime \prime \prime } dV$$

Finally, the total entropy generation rate can be expressed as follows:

17 $$S_{Total} = \dot{S}_{{\overline{D}}} + \dot{S}_{{D^{\prime}}} + \dot{S}_{w}$$

### Geometric model

#### Axial-flow pump model

**Figure 4:**
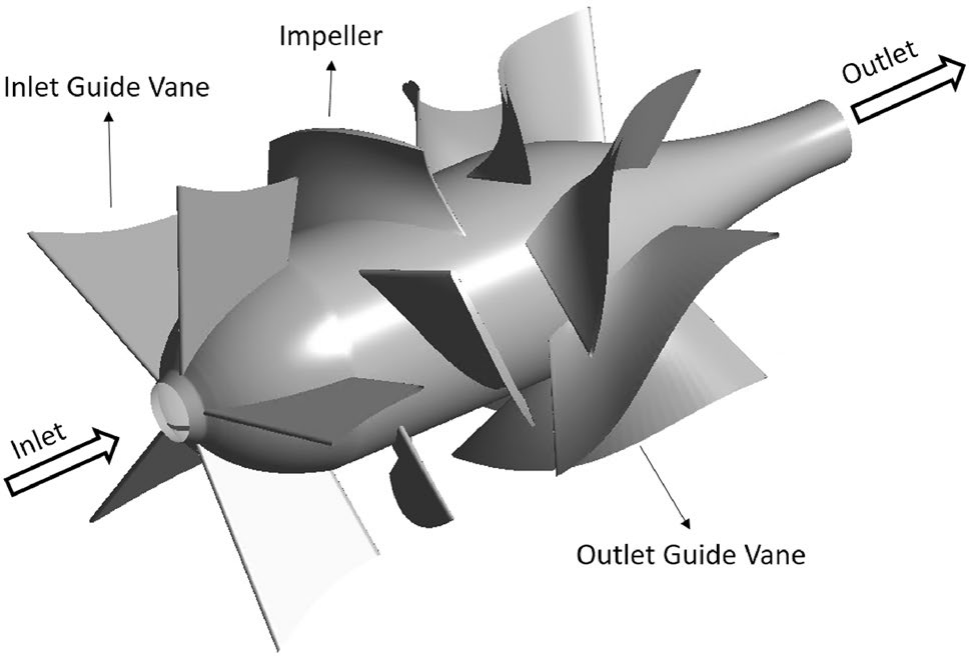
Axial-flow pump model.

The axial-flow pump, with a specific speed of 1204, determined using Eq. (18), is the research object of the present work. Figure 4 presents the three-dimensional geometry of the axial-flow pump, which includes five stationary IGVs, four rotating impellers, and seven stationary DVs. Using the similarity law^43^, the real model is scaled down by a factor of eight from the original. The inlet and outlet regions are expanded to nearly four and five times the impeller’s diameter, respectively, to avoid interference with the fluid flow field, whereby the total development of the turbulent flow could be examined. Table 1 presents some main parameters of the pump model. The design discharge rate coefficient (Eq. (19)), total head coefficient (Eq. (20)), and rotating speed coefficient (Eq. (21)) are 0.4319, 1.5841, and 0.7893, respectively. To observe and describe the asymmetries of the real internal fluid flow field, it is ideal to model all passages in the computational domain. Nevertheless, most numerical calculations of the fluid flow in axial turbomachinery use the benefit of the periodic geometry. In the present study, only one IGV, one impeller, and two DV passages with a pitch ratio of 0.8: 1.00: 1.14 are modeled in the computational domain. The pitch ratio should be near 1.0 to improve the calculation accuracy and reduce profile scaling^44^. One IGV and two DV passages are, thereby, represented in the computational domain.

18 $$\lambda = n\left[ {rpm} \right]\frac{{\varphi^{0.5} \left[ {\frac{{m^{3} }}{min}} \right]}}{{\psi^{0.75} \left[ m \right]}}$$

19 $$H = \frac{\varphi }{{nD^{3} }}$$

20 $$Q = \frac{g\psi }{{n^{2} D^{2} }}$$

21 $$\chi = \frac{nD}{{\sqrt {g\psi } }}$$

where $n$, $\varphi$, $\psi$, $D$, and $g$ represent the rotating speed, discharge rate, pressure head, impeller diameter, and gravity acceleration, respectively.

**Table 1:** Parameters of the axial-flow pump model.

Parameter	Value
Specific speed	1204
Rotating speed coefficient	0.7893
Design head coefficient	1.5841
Design discharge rate coefficient	0.4319
Number of IGV blades	5
Number of impeller blades	4
Number of DV blades	7

### New IGV design

The new IGV design is shown in Figure 1c. The NACA foil profile was selected as the new hydrofoil for the IGV. The NACA foil profiles are characterized by a particular shape that offers a suitable lift-to-drag ratio, resulting in an improvement in energy performance over that of other airfoil types. The profile is described by a set of numerical values that determine the camber (curvature) and thickness of the airfoil at different points. The hydrofoil profile of the IGV can be classified into two forms: symmetric and asymmetric. In this study, the reference and new designs of the IGV are symmetric and asymmetric, respectively, with the NACA 2412 airfoil. The thickness of the new IGV is different at each span, whereas that of the reference IGV is the same. The thickness of the reference IGV is equal to 0.013 times the impeller's diameter. Meanwhile, the thickness of the new IGV is gradually reduced from the shroud to the hub, with thicknesses at the shroud, mid-span, and hub of 0.043, 0.037, and 0.032 times the impeller's diameter, respectively. With this change in thickness, manufacturing the rotating mechanism of the IGV^6^ becomes easier in the future, producing a stronger product with a large thickness at the shroud. In addition, for the convenience of fabrication, the TE of the IGV is also rounded with an elliptical ratio of 1. The blade angle at the TE of the new IGV is also different from that of the reference IGV, whereas the blade angle at the LE is the same. The blade angle at the TE of the new IGV is fabricated to decrease from the shroud to the hub, as shown in Figure 1c, to create a smooth velocity profile at the LE of the impeller. In this study, the new IGV model is defined as a model consisting of the new IGV, a reference impeller, and reference DV geometries.

#### New DV design

**Figure 5:**
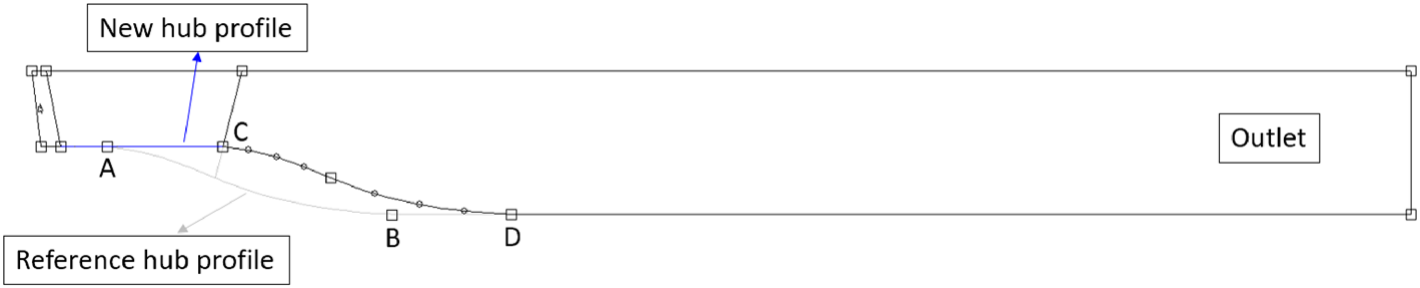
Hub profile of the DV.

The Figure 5 presents a new DV with a change in the hub profile. In the present study, the hydrofoil profile of the new DV and that of the reference DV are the same, whereas the hub profile of the new DV is straightened from the LE to TE. This creates a new DV, with the hub and shroud profiles parallel to the axis of rotation. The hub profile behind the TE from C to D of the new DV is designed to be similar to the hub profile from A to B of the reference DV by the translation method. After the change, the cross-sectional area inside the new DV passage is constant, whereas that of the reference DV gradually increases. In this study, the computational model, which includes the new IGV geometry, reference impeller, and a new DV hub profile is referred to as the new IGV and DV model.

#### Adjustable DV

The new IGV geometry and new DV hub profile are used in the computational models of the rotating DV. Figure 2 shows a two-dimensional design of the DV. The rotation shaft is created by a line connecting the middle point of the camber line at the hub and shroud of the DV profile. To investigate the influence of different rotation positions, six different setting angles of the DV are tested: $\pm 5^\circ$, $\pm 10^\circ$, and $\pm 12.5^\circ$. Here, the setting angle corresponding to clockwise rotation is defined as positive, whereas that corresponding to counterclockwise is negative. The reference position of the DV is considered to be the $0^\circ$ setting angle of the DV.

#### Adjustable IGV and DV

After the results for different DV setting angles are obtained, a numerical scheme for the combinations of simultaneous rotating IGV and DV is established. The effect of different IGV setting angles on the energy performance of the axial-flow pump was analyzed in detail in a previous study^6^. In the present work, taking advantage of the rotating IGV, the combinations of simultaneously implemented adjustable IGV and DV are classified into three main cases. In the first case, the IGV setting angle is set to $15^\circ$, and the DV setting angle is $10^\circ$. In the second case, the setting angles of the IGV and DV are set to $15^\circ$ and $-10^\circ$, respectively. Finally, the angles are set to $-15^\circ$ and $-10^\circ$, respectively. A 2D drawing of the axial-flow pump with adjustable IGV and DV is illustrated in Fig. 2.

### Numerical analysis

#### Boundary conditions

The initial and boundary conditions of the computational domain are established by using CFX-Pre supplied by ANSYS, with a working fluid set to water at 25 °C. Adiabatic and no-slip conditions at the walls are employed for the blades, hub, and shroud. A periodic condition is applied at the sides of each channel. The interfaces between the stationary and rotating frames are connected using the stage model. At the inlet and outlet of the axial-flow pump, the total pressure and mass flow rate are set, respectively. In the rotating impeller, the alternative rotational model is activated to decrease the generation of false swirls, as the mesh systems are too rough and/or the fluid flow movement is not properly matched with the rotational frame. Using an intersection algorithm^44^, the General Grid Interface is used to transmit data between the intersections of the frames. In turbomachinery, a physical time scale (Eq. (22)) is often used to accelerate the convergence time^45^. To verify the convergence simulation, in the time-dependent convergence standard, the root-mean-square values of the residuals are established at 10^−6^, and the deviation from balance in the mass is below 1%.

22 $$\xi = \frac{1}{10\delta N}(s)$$

where $\delta$ stands for the impeller angular velocity and $N$ is the number of impeller blades.

The unsteady simulations are initialized by using the steady results to reduce the convergence time. For the dynamic–static interfaces, a transient rotor stator model is adopted. The time step is set to approximately $2\times {10}^{-4}$ s, which is the rotation time for the $3^\circ$ rotating impeller. The total simulation time is set to 10 times the impeller's rotation cycle ($\zeta$), at approximately 0.234 s. For further convergence, four coefficient loops are set for each time step.

#### Mesh generation and independence test

**Figure 6:**
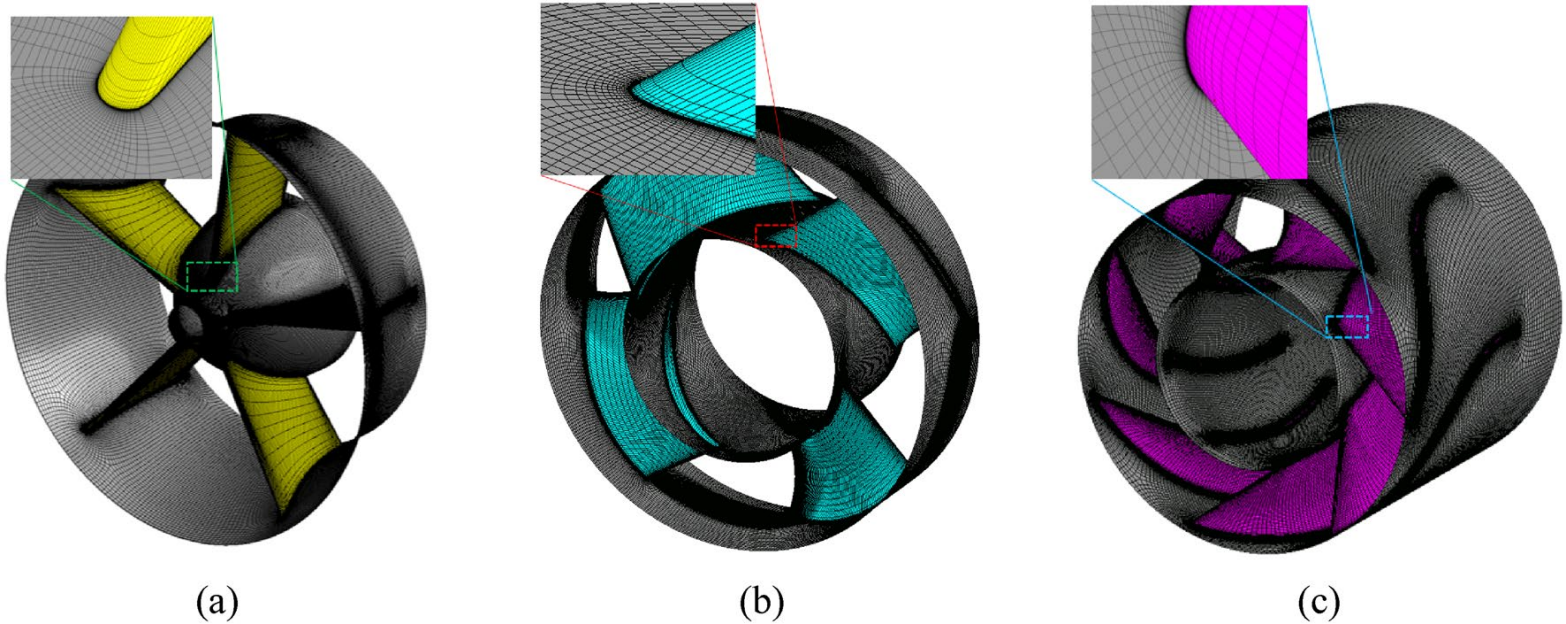
Grid systems in numerical simulation. (**a**) IGV, (**b**) Impeller, and (**c**) DV.

The hexahedral mesh systems are created using the TURBOGRID supplied by ANSYS commercial code software. The mesh systems of the axial-flow pump are presented in Figure 6. To obtain the y^+^-averaged values over walls less than two, numerous layers of prism mesh are created at these wall areas with small thickness. The mesh independency is sequentially checked at the impeller, IGV, and DV passages. Consequently, after the optimum impeller mesh is acquired, it is further used to obtain the optimum IGV mesh. Then, with optimal meshes for the impeller and IGV, the optimal mesh structure of the DV is found.

The convergence time, discretization error, and precision of the results are all greatly influenced by the quality of the mesh system. Furthermore, the computing power is directly affected by the number of nodes. Therefore, examining mesh independence is essential to determine the optimum mesh structure. As demonstrated in a previous study^6^, the independence of the mesh system can be verified by using the grid convergence index scheme (GCI), which was created by Celik^46^. The GCI scheme estimates the numerical uncertainty arising from discretization errors, using three distinct meshes (coarse, medium, and fine meshes). The extrapolation is performed using a grid refinement factor greater than 1.3. The BEP is selected to verify the grid’s independence. The key variable used in the test procedure is the efficiency (η). With the increase in the number of nodes, the efficiency values in Table 2 demonstrate a monotone convergence. The difference between the efficiency values becomes comparatively small as the number of nodes increases. The index values of the important variables should be lower than 1%, according to Celik^46^. Consequently, the low values of the GCI (${GCI}_{fine}^{21}$) and extrapolated relative error (${e}_{ext}^{21}$) show that the developed mesh schemes are optimal and do not require further mesh refinement. The optimal numbers of nodes for the single IGV, impeller, and DV passages are 0.55 × 10^6^, 0.67 × 10^6^, and 0.57 × 10^6^, respectively.

**Table 2:** Mesh independence analysis^6^.

	Only impeller (No. of nodes in impeller)	IGV and impeller (No. of nodes in IGV)	Full component (No. of nodes in DV)
N_1_	666,773	550,272	571,540
N_2_	265,681	248,100	256,250
N_3_	115,260	409,164	114,500
r_21_	1.359	1.304	1.307
r_32_	1.321	1.315	1.308
${\upeta }_{1}/{\upeta }_{1}$	1.00	0.976	1.043
${\upeta }_{2}/{\upeta }_{1}$	0.993	0.975	1.042
${\upeta }_{3}/{\upeta }_{1}$	0.972	0.957	1.026
p	3.769	9.199	9.123
${e}_{ext}^{21}$	0.00325	0.00014	0.00012
${{\text{GCI}}}_{medium}^{32}$	0.01412	0.00197	0.00178
${{\text{GCI}}}_{{\text{fine}}}^{21}$	0.00407	0.00017	0.00016

#### Numerical validation

**Figure 7:**
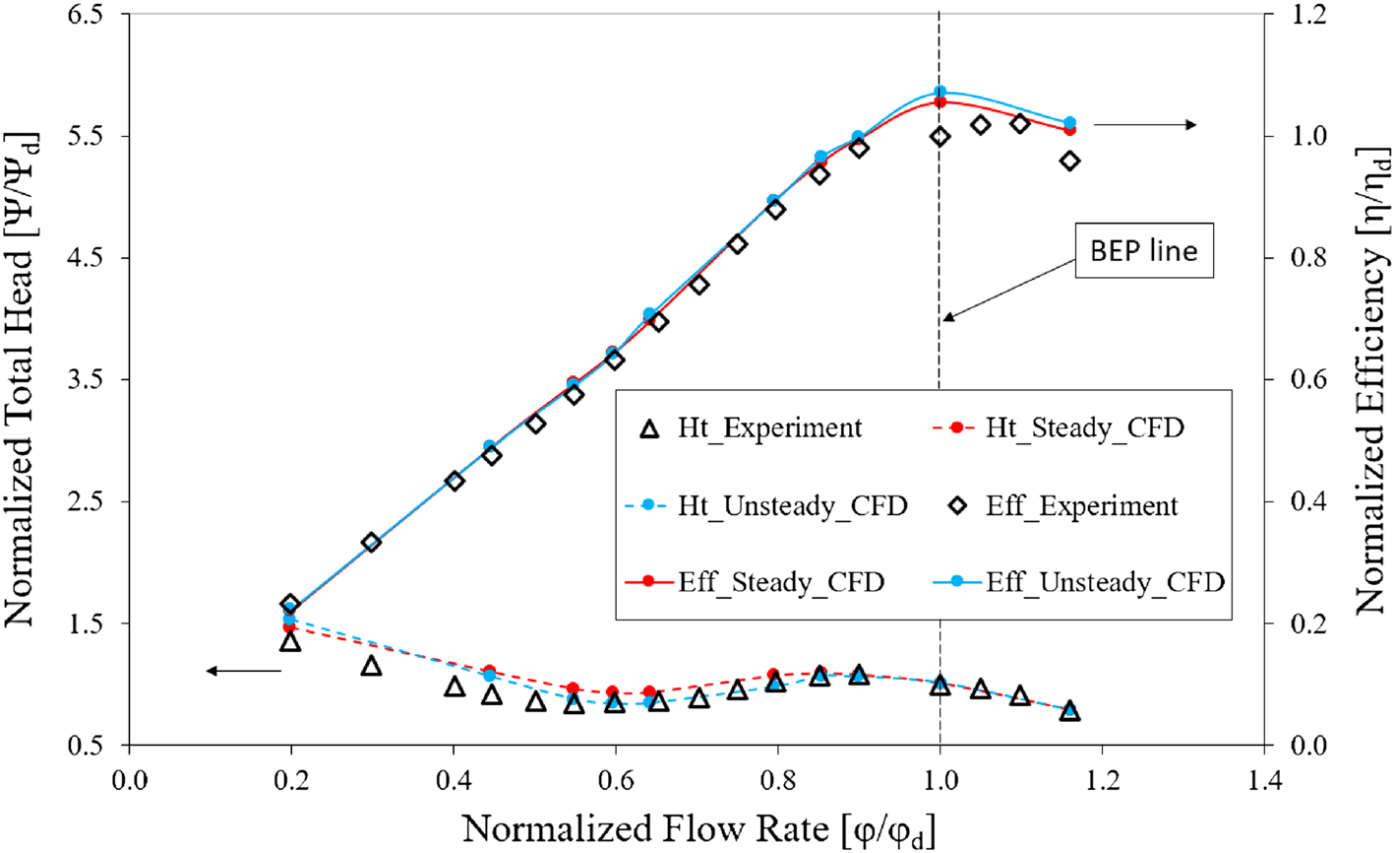
Energy performance curves in the numerical and experimental test.

A comparison between the experimental and numerical results of the axial-flow pump is presented in Fig. 7. The efficiency and total head values are normalized by their respective values under the design conditions of the experiment. As analyzed thoroughly in the validation section of the previous study^6^ and presented in Fig. 7, the obtained numerical results show good agreement with the experimental results. At the BEP, the efficiency of the numerical calculation is just 5.2% greater than that of the experiment, while the pressure head of both is nearly identical. The differences in the results are caused by measurement errors, instrument uncertainty, mechanical loss, and errors in numerical simulations. In the high-flow-rate region (larger than 1.2 ${\varphi }_{d}$), the experimental results are unavailable, as the experimental test yielded large hydraulic loss owing to long pipelines and multiple couplings without the use of a booster pump. The hydraulic loss generated under the high-flow-rate condition is relatively large including the cavitation problem which results in the complex flow field. For this reason, the experimental results are a little unstable from the BEP to the high-flow-rate region resulting in a large difference of the efficiency values. The trend of the unsteady results is consistent with those of the steady and experimental results. In the saddle zone^6^, the unsteady results show better agreement with the experiment than do the steady results, as the calculations are more accurate in the unstable cavitation phenomena. Consequently, the numerical results in this study are reasonable and reliable in comparison with the experimental results.

## Results and discussion

### Effect of the new IGV profile

**Figure 8:**
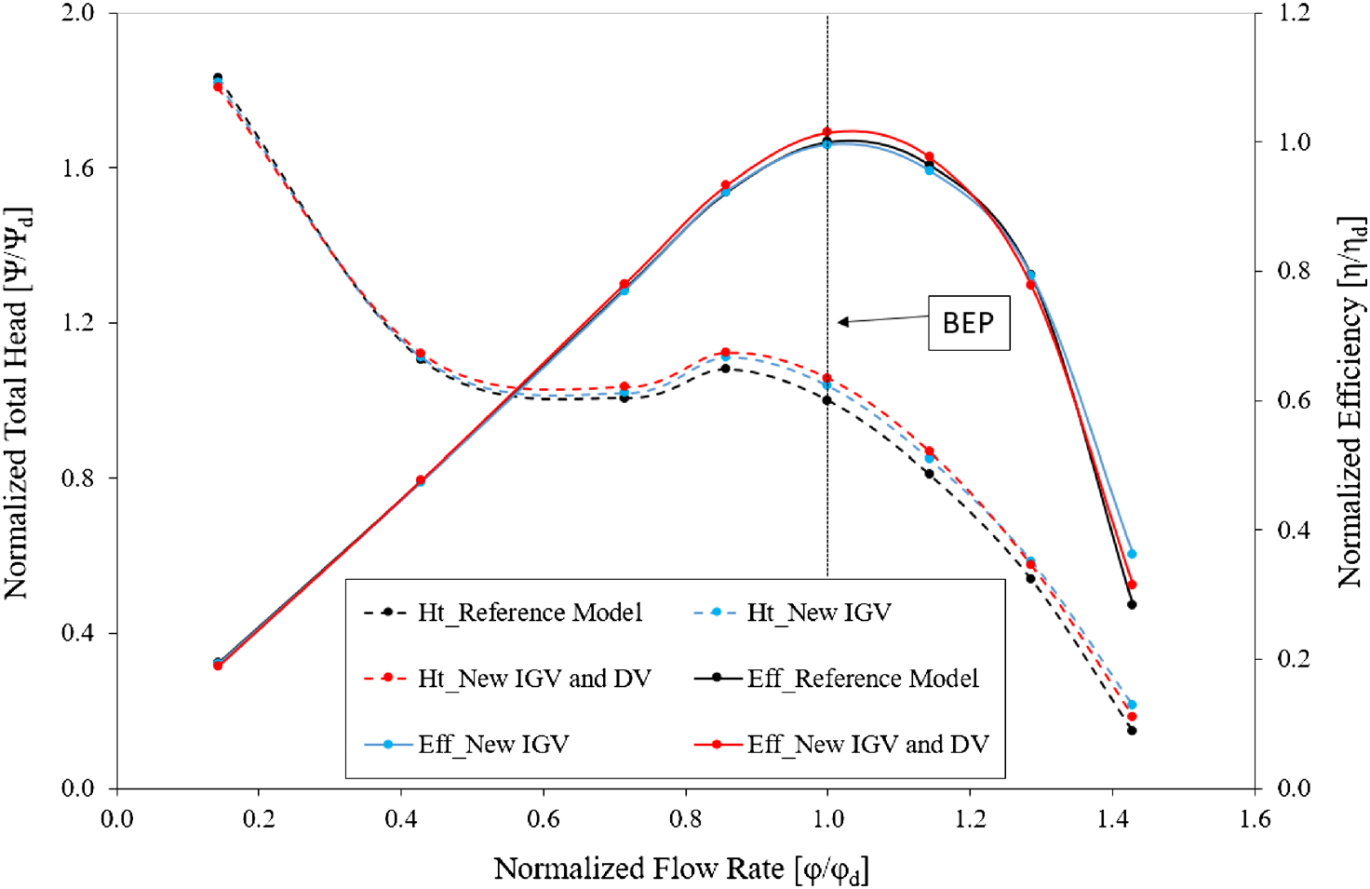
Energy performance curves of the reference and new models.

The Fig. 8 shows the energy performance of the model pump for the reference model, new IGV model, and new IGV and DV model. The total efficiency and total head values are extracted at inlet and outlet of the axial-flow pump and they are normalized using their respective values under the design conditions of the reference model. Operating with the new IGV, the energy performance changes significantly, with a sharp enhancement in the total pressure at all operating points and an increase in efficiency in the high-flow-rate region. The improvement in the total head is stable and follows an increasing trend from the low-flow-rate region near 0.86 ${\varphi }_{d}$ to the high-flow-rate region, with an increase of at least 2.89% compared to the total head of the reference model. This indicates a steady improvement in the new IGV under both low-flow-rate and high-flow-rate conditions. Under the design conditions, although the efficiency does not increase, the total head value increases up to 3.75% in comparison with that of the reference model. This represents a major contribution to the performance of the axial-flow pump. More specifically, at the flow rate of 1.43 ${\varphi }_{d}$, the efficiency and total head are significantly greater, up to 27.75% and 44.77%, respectively, than the reference model. This shows that the operation of the new IGV has a positive effect under the high-flow-rate conditions, in which the axial-flow pump often operates. In addition, the total head does not significantly increase in the low-flow-rate region; however, it does slightly improve compared to that of the reference model. Consequently, this also provides a positive impact on the cavitation phenomena if it occurs in the saddle zone^6^ of the axial-flow pump.

**Figure 9:**
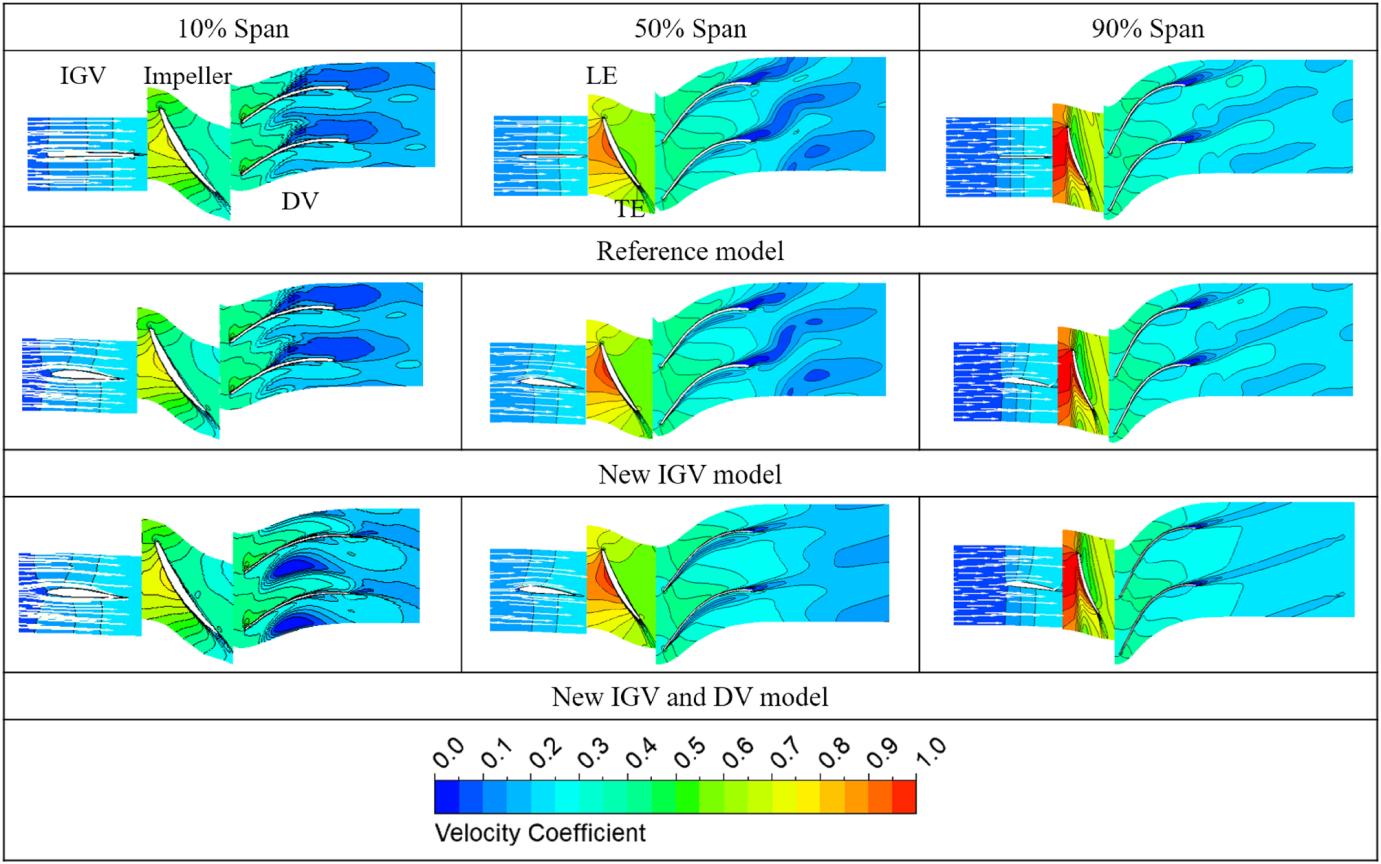
Velocity distributions in the three spans at the BEP.

The Figure 9 presents the velocity distribution among the three models at three different spans in the axial-flow pump at the BEP. The velocity coefficient is determined by its ratio to the velocity at the impeller tip. The operation of the new IGV with a higher blade angle reduces the absolute flow angle at the LE of the impeller, facilitating the high-energy fluid flow into the impeller. This can be clearly seen by comparing the direction of the velocity vectors of the reference and new IGV models in the IGV passage. In the reference model, the direction of the velocity vector after passing through the IGV is straight, whereas that of the new IGV tends to point in the direction opposite to that of the impeller rotation. An improvement in the fluid flow energy is also evident at the SS of the impeller with increase in velocity. In addition, the velocity distribution in the PS of the impeller also becomes more even than that of the reference model with the elimination of low-velocity at a position of approximately 60% of the chord line. The improvement in the velocity distribution in the impeller passage contributes significantly to the increase in the flow energy, enhancing the total head of the axial-flow pump.

**Figure 10:**
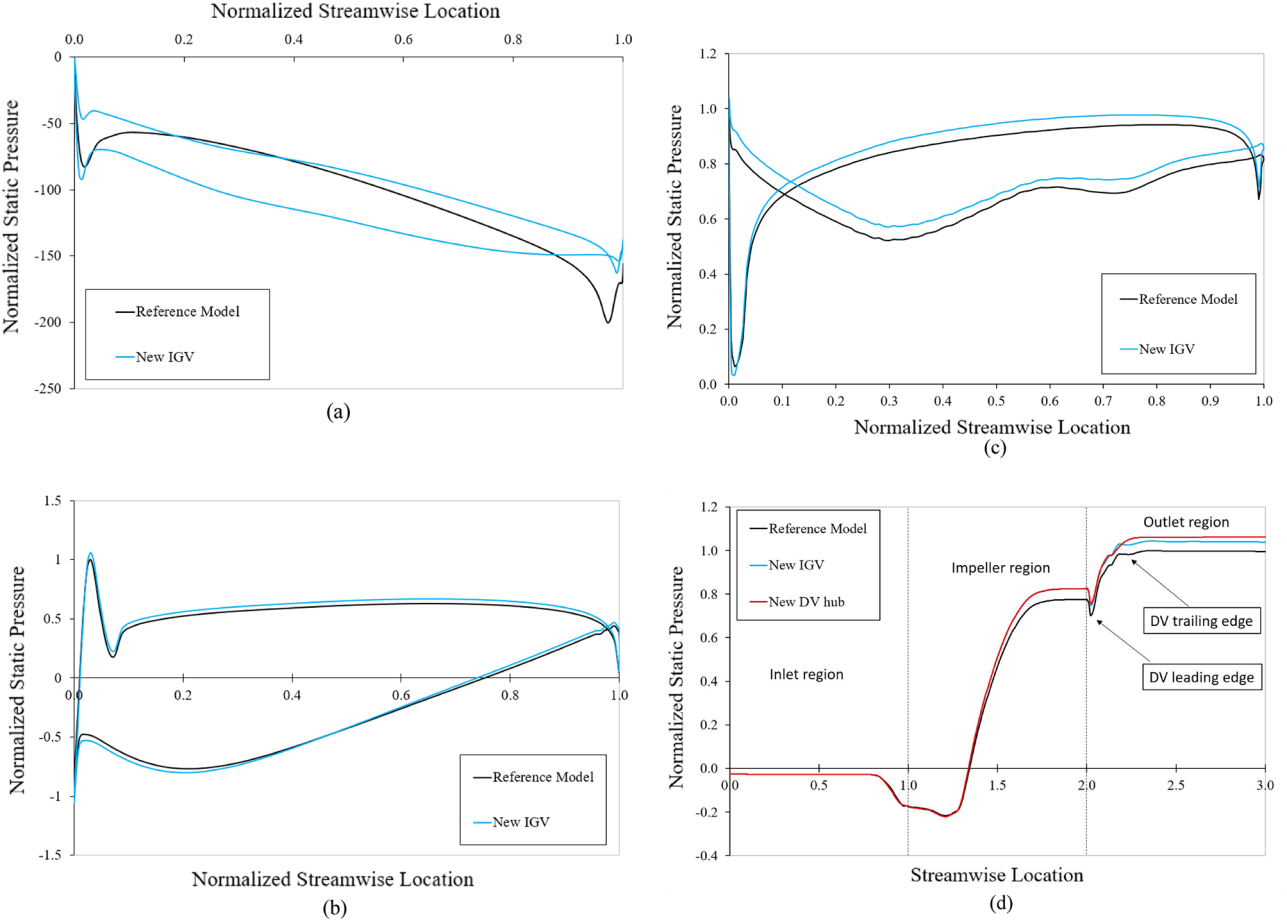
Pressure distribution at the BEP. (**a**) 50% span of the IGV, (**b**) 50% span of the impeller, (**c**) 50% span of the DV, (**d**) From inlet to outlet.

To clearly reveal the improvement in the total head of the axial-flow pump, the pressure distributions at 50% span of the IGV, impeller, and DV at the BEP are depicted in Figure 10. The pressure values are non-dimensionalized, based on the highest pressure value at each part of the reference model. A clear difference between the reference IGV and new IGV is shown in Figure 10a. The pressure distribution of the reference IGV has only one line because of the symmetry in its geometry, resulting in the same pressure distribution on both sides. With the new IGV using a hydrofoil profile, the fluid flow passes through IGV with extra energy owing to the differential pressure between the PS and SS, which is clearly shown in Figure 10a. In addition, there is a considerable increase in the pressure distribution at the TE of the new IGV, which improves the cavitation if present. Furthermore, this also increases the inlet pressure of the impeller and causes the pressure at the impeller to increase accordingly. This can be clearly observed at the PS of the impeller, with a steady increase in pressure across each streamwise position. Likewise, the flow energy obtained from the impeller continues to improve significantly at DV, increasing the total head of the axial-flow pump. There is a sudden increase in the pressure distribution at a streamwise position of around 0.03 of the impeller, owing to the presence of the stagnation point at the LE, where the fluid flow comes into direct contact. Additionally, because of the separation flow at TE^47^, the last streamwise position, the pressure distribution decreases strongly, not only in the impeller but also in both the IGV and DV. This can be clearly seen in Figure 9, in which the velocity notably decreases at the TE of each part, causing chaos and hydraulic loss in the fluid flow. Figure 10d presents the averaged pressure distribution from the inlet to the outlet of the axial-flow pump among the three models at BEP. The pressure increases sharply in the impeller region because the fluid flow receives a large energy from the rotating impeller. Clearly, with the new IGV, the pressure increases sharply in the impeller region and continues to maintain an upward trend in the DV region. The uptrend of pressure in the impeller is uniform and consistent, showing stability during the operation of the new IGV. Consequently, using the hydrofoil with NACA profile in the IGV has a significantly positive effect over the performance of the reference IGV using symmetrical geometry.

**Figure 11:**
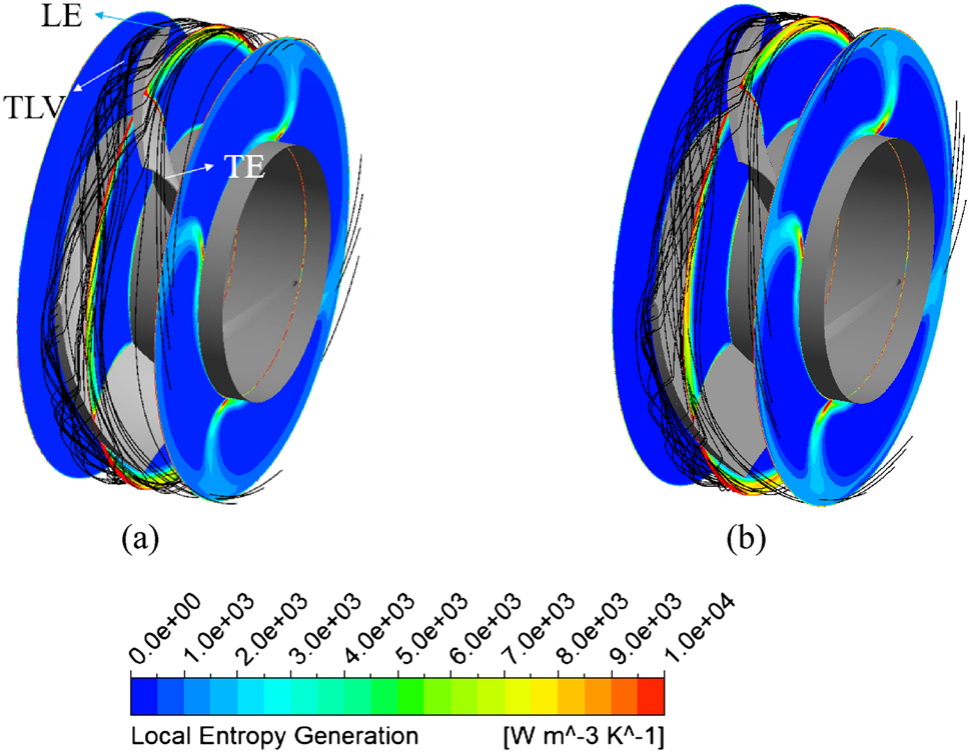
Local entropy generation and streamline distributions in the impeller passage at the BEP. (**a**) Reference model, (**b**) New IGV model.

The Figure 11 shows the local entropy generation in the three planes located in the inlet, middle, and outlet of the impeller passage at the BEP. In the inlet plane, entropy does not seem to be significant, as the flow after passing through the IGV is smooth and steady in both models. In the middle plane, a large amount of entropy is generated and concentrated mainly at the SS of the impeller’s tip region owing to the presence of a tip leakage vortex (TLV)^25^, which causes the flow to be more chaotic. Further away from the SS of the impeller, the local entropy generation is reduced because of the weakening of the TLV. This indicates that the TLV plays a significant role as the primary reason for the energy loss of the flow. In the outlet plane, a small amount of entropy is produced at the TE of the impeller near the hub region owing to the disturbance of the flow, induced by the flow separation at the TE of the impeller, as mentioned above. Evidently, there is no significant impact from visual observation of Figure 11 in the entropy generation occurring at the impeller passage by changing the geometry of the IGV. However, compared to operating with the reference IGV at the BEP, the local entropy generation in the impeller passage when operating with new IGV is increased by 22.1% while it is decreased by 4.9% and 1.29% in the IGV and DV passages, respectively. The reason for the increase of the local entropy generation in the impeller passage can be attributed to the increase in the intensity of the turbulent flow caused by the acceleration in the velocity field as shown in Figure 9. Thus, based on the local entropy generation, it can state that the energy dissipation in the impeller when operating with the new IGV is slightly increased in comparison with that of the reference IGV.

#### Effect of the new DV hub profile

As shown in Fig. 8, the combination of the new IGV and new DV presents energy performance that is clearly improved over those of both the reference and new IGV models. Around the BEP region, the energy performance increases notably in both the efficiency and total head. The standout highlight of the new DV design is the improvement in the efficiency at the BEP, exhibiting an increase in the efficiency and total head of 1.914% and 1.93%, respectively, compared to those of the new IGV model. Therefore, the effect of the DV hub profile on the energy performance is considerable. Although the efficiency and total head increase progressively from the saddle zone to the high-flow-rate region, they remain less than those of the new IGV model at a flow rate of around 1.143 ${\varphi }_{d}$. However, the energy performance is still maintained at a higher level than that of the reference model. In addition, the reduction in energy performance under high-flow-rate conditions can be improved by adjusting the DV, which is discussed in the next section. Through changes in the IGV geometry and hub profile of the DV, the efficiency and total head of the axial-flow pump at the BEP are considerably improved, compared to those of the reference model, by 1.456% and 5.756%, respectively.

**Figure 12:**
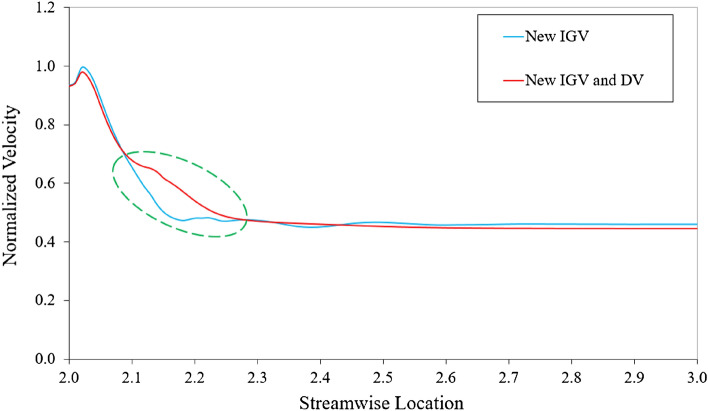
Velocity distributions in the DV passage at the BEP.

The Figure 12 shows the average velocity distributions from the inlet of the DV passage to the outlet for the new IGV model and new IGV and DV model at the BEP. The velocities are normalized using the highest velocity value in the DV passage of the new IGV model. The gradual decrease of velocity in Figure 12 and the gradual increase of pressure in Figure 10d in DV passage clearly demonstrate the role of DV in the energy conversion from kinetic energy to pressure energy. Evidently, there is a difference in the velocity distributions of the two models from a streamwise location of 2.1 to around 2.2, where the hub profile changes. The increase in the cross-sectional area inside the DV passage causes a rapid and strong reduction in the velocity of the new IGV model, which easily leads to the formation of an adverse velocity gradient and turbulence, causing energy loss. This can be observed in the distribution of the velocity from a streamwise location of around 2.2 to 2.6, the velocity distribution is not as stable as that of the new IGV and DV model. In addition, Figure 9 also shows that there is a low-velocity zone formed at the TE region of the DV, causing energy dissipation. Therefore, increasing the cross-sectional area inside the DV passage in this axial-flow pump model is detrimental to energy performance. With the new DV hub, the velocity decreases slowly and becomes stable as soon as the cross-sectional area becomes constant from around streamwise location of 2.3 to the outlet. This reduces hydraulic loss by improving turbulent flow at the TE of the DV, as shown in Figure 9, leading to an improvement in energy performance. At 10% span, a low-velocity zone forms in the passage of the new IGV and DV model. This low-velocity zone causes an insignificant loss of energy and is analyzed in more detail below.

**Figure 13:**
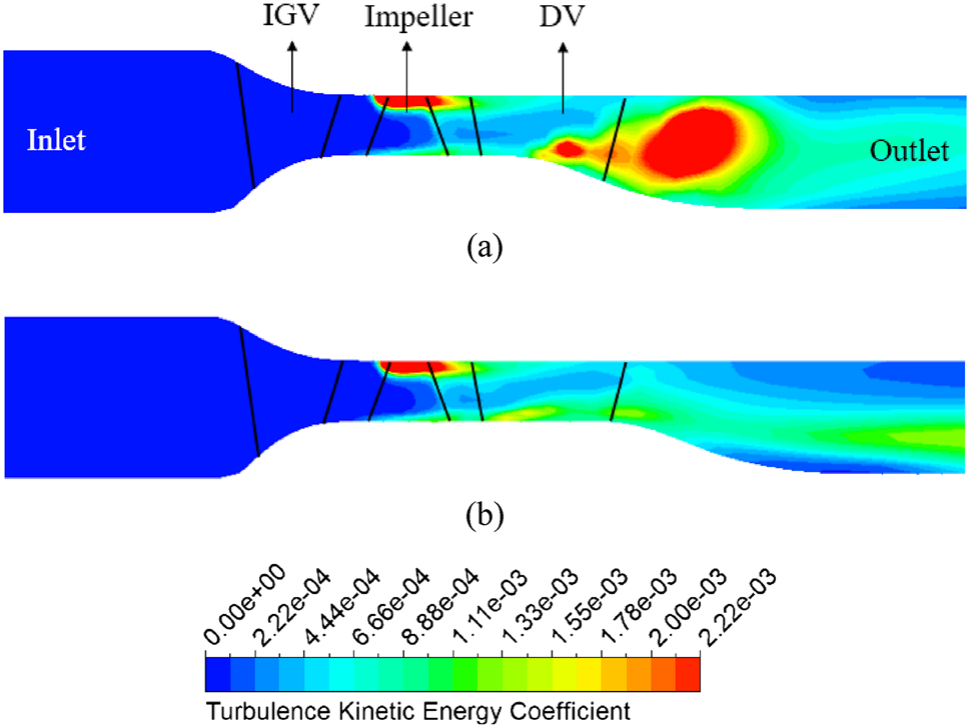
TKE in the meridional plane at the BEP. (**a**) New IGV, (**b**) New IGV and DV.

To clearly demonstrate the intensity of turbulence energy generated in the DV passage, Figure 13 presents the TKE along the meridional plane between the new IGV model and new IGV and DV model at the BEP. The TKE coefficient is defined by its ratio to the square of the velocity at the impeller’s tip. In the new IGV model, the increase in the cross-sectional area right at the middle of the DV passage immediately causes the appearance of a low-velocity region resulting in turbulent flow, as shown in Figure 9 at 10% span. This amount of turbulence strongly propagates in the DV passage, producing a large amount of TKE, which leads to large energy loss. By changing the hub profile, there is a clear improvement in the velocity field around the TE region of the DV, as the low-velocity region is reduced, leading to less TKE than what is found in the new IGV model. As shown in Figure 13b, the energy loss in the low-velocity region at 10% span of the new IGV and DV model is negligible, accompanied by low TKE over a small area. Therefore, it is evident that the energy performance of the axial-flow pump is increased by suppressing the turbulent flow behind the TE of the DV.

**Figure 14:**
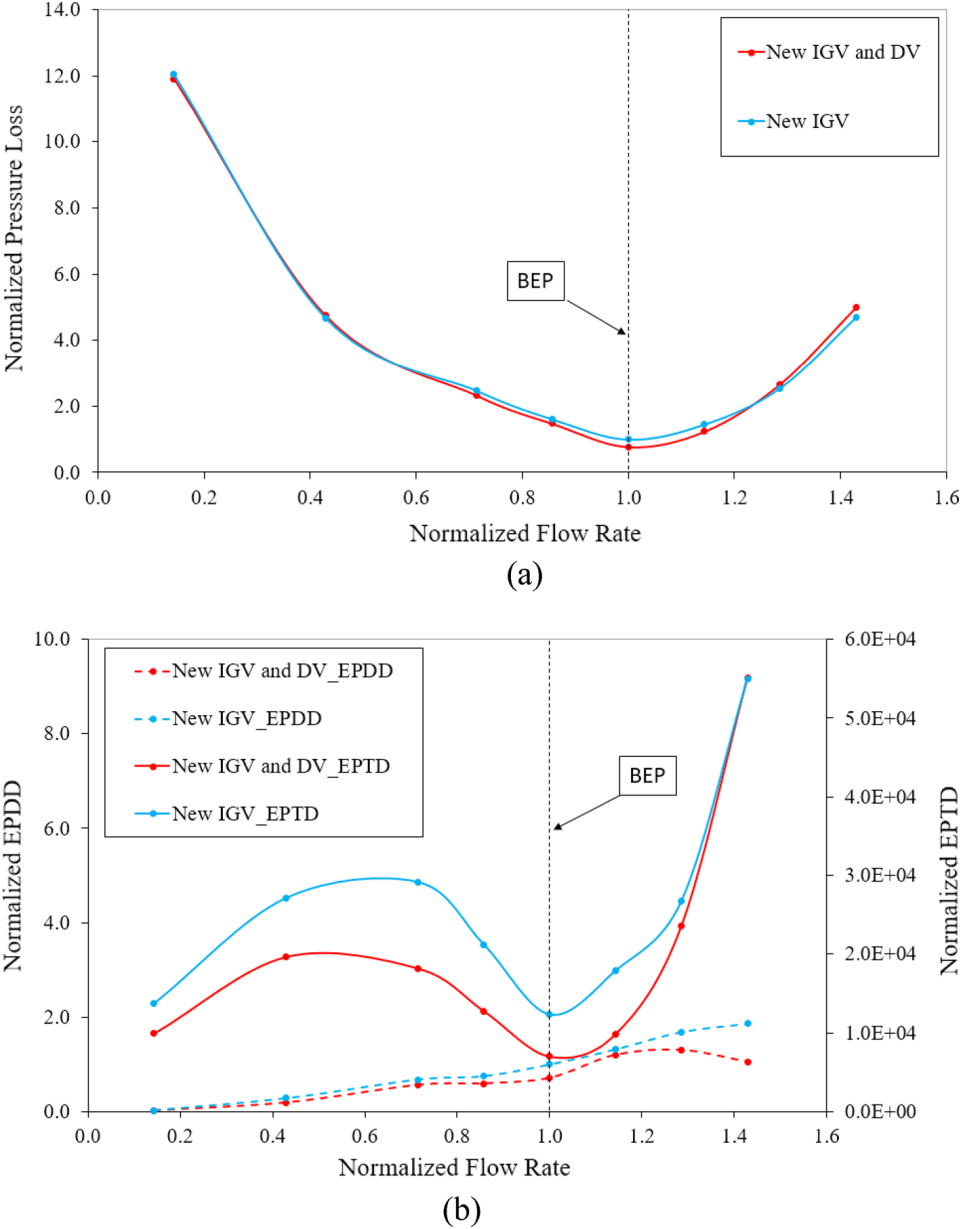
Energy losses in the DV passage. (**a**) Pressure loss and (**b**) Local entropy generation.

The Fig. 14 depicts the pressure loss and local entropy generation in the DV passage under different flow rates for the new IGV model and new IGV and DV model. The pressure loss is normalized, using its value at the BEP of the new IGV model. Likewise, the EPDD and EPTD are normalized based on the value of the EPDD at the BEP of the new IGV model. From Fig. 14a, the pressure loss curves tend to be inversely similar to the efficiency curve with the minimum pressure loss at the BEP, whereby it increases with increasing or decreasing flow rate. This indicates a close relationship between pressure loss and pump performance. At the BEP, with an increase in the efficiency, the pressure loss of the new IGV and DV model is reduced by 22.76% compared to that of the new IGV model. The trend of the EPTD is quite similar to that of the hydraulic loss, except that the flow rate is less than 0.714 ${\varphi }_{d}$. As shown in Fig. 14b, the trend of the EPDD is a gradual increase from low-flow-rate to high-flow-rate. As the EPDD is directly affected by the velocity gradient, when the flow rate rises, the average velocity also increases, facilitating the momentum transfer between fluid layers. This ultimately leads to heat generation from viscous friction, which gradually increases the EPDD. Local entropy generation caused by direct dissipation and by turbulent dissipation is basically a process of converting the kinetic energy of the fluid flow into thermal energy through viscosity and turbulence, respectively. As shown in Fig. 14b, the local entropy generation caused by the EPDD is much smaller than that caused by the EPTD. This is due to the fact that turbulent flow can more effectively transform its energy into thermal energy across a much wider range of scales than viscosity can, increasing the local entropy generation by the EPTD. Therefore, it can be concluded that the local entropy generation in the DV passage is mainly caused by the EPTD. The EPTD of the new IGV and DV model is notably decreased in comparison with the new IGV model, except at the flow rate of 1.43 ${\varphi }_{d}$ due to high turbulent flow. At the BEP, the EPTD of the new IGV and DV model is less than that of the new IGV model, by 43.38%, owing to the improvement in the turbulence in the DV passage. The EPTD is elevated in the saddle zone owing to the existence of cavitation, resulting in a significant disturbance in the fluid flow.

**Figure 15:**
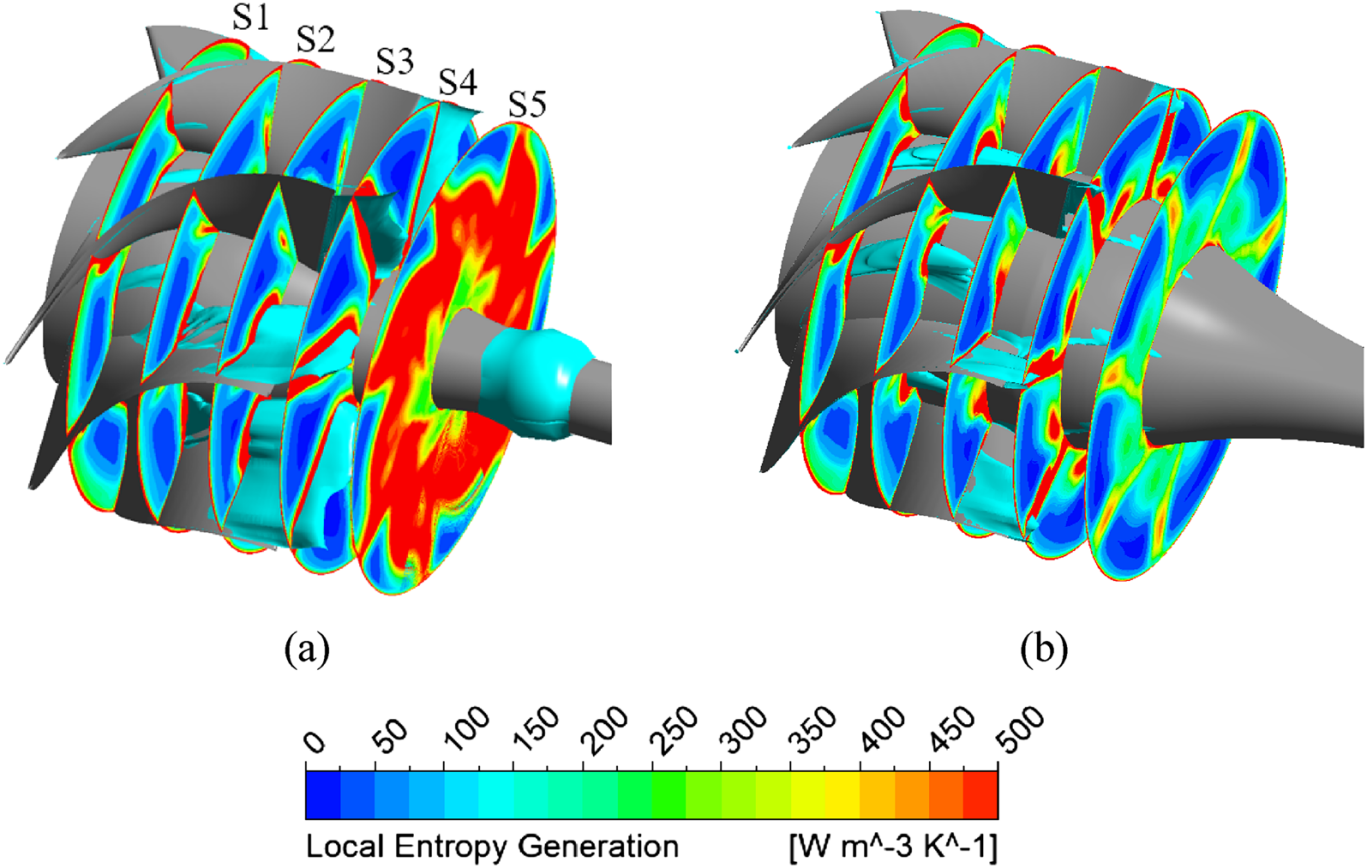
Distribution of the local entropy generation and low-velocity zone in the DV passage at the BEP. (**a**) New IGV, (**b**) New IGV and DV.

The Figure 15 presents the local entropy generation across the cross-sections in the DV passage for the new IGV model and new IGV and DV model at the BEP. Meanwhile, the three-dimensional distribution of the low-velocity zone in the DV passage, with a velocity coefficient of 0.08, is also shown in cyan. As can be seen, the low-velocity zone distributed in the DV is associated with the region of high entropy generation. This shows that, at the BEP, low-velocity zones generate a significant amount of turbulent kinetic energy, leading to energy dissipation and reduced efficiency. The low-velocity zone of the new IGV model is formed by flow separation at the SS of the DV, starting at plane S2 and extending through plane S4, causing large energy loss. This low-velocity zone is clearly improved in the new IGV and DV model, whereby, the entropy generation caused by turbulent flow at plane S4, and especially plane S5, is drastically reduced. In the wall regions, such as the shroud, hub, and blade surface, the entropy generation is quite high, as the friction of the fluid flow and wall generates heat, causing energy loss. In addition, the entropy generation caused by direct dissipation is mainly concentrated in these wall regions. In the hub region of the new IGV and DV model, a small amount of entropy generation is evident, caused by the low-velocity zone propagating from plane S1 to near S3. At plane S4, although the entropy generation is notably reduced, owing to the removal of the low-velocity zone, a significant amount of entropy is still generated owing to the flow separation at the TE of the DV^47^. This may be unavoidable for fluid flow in turbomachinery. In addition, the low-velocity zone of the new IGV model present right behind plane S5, owing to the enlarged cross-sectional area in the DV passage, is also eliminated in the new IGV and DV model.

**Figure 16:**
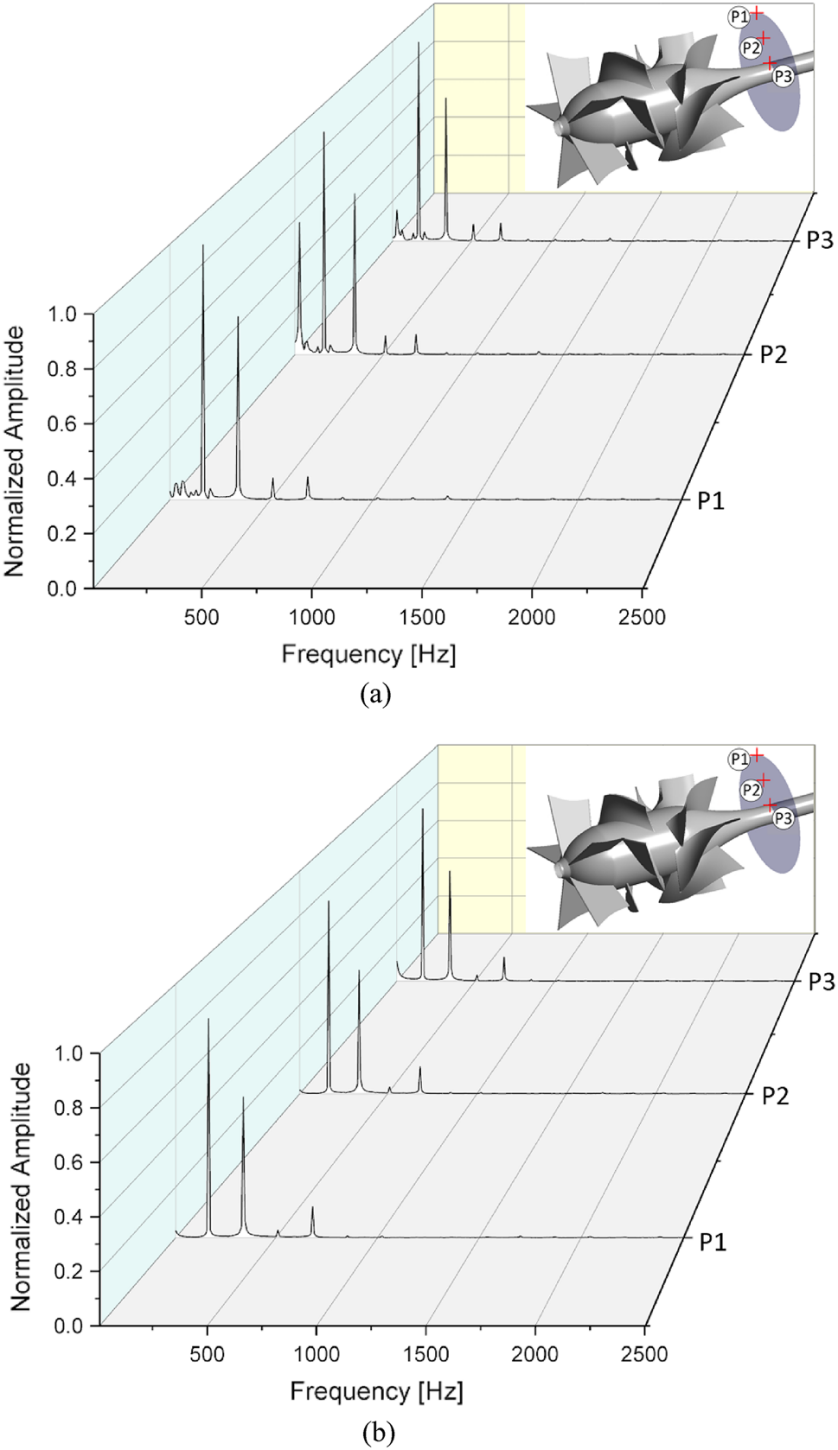
Amplitude spectra of monitoring points at the BEP. (**a**) New IGV, (**b**) New IGV and DV.

With respect to the vibration and noise in the pump, Figure 16 shows the frequency spectra of the amplitude at the monitoring positions for the new IGV model and new IGV and DV model at the BEP. The monitoring positions are set behind the DV, where the cross-sectional area remains constant, to obtain stable flow. Therefore, the amplitudes at all three positions are almost the same. The fast Fourier transformation (FFT) algorithm^48^ is used to create the frequency spectra based on the unsteady pressure fluctuation with time. Amplitude is normalized based on the maximum amplitude value among the monitoring positions of the new IGV model. Based on the number of impeller blades and rotational speed, the blade passing frequency (BPF) is roughly 170.67 Hz (Eq. (23)), which corresponds to the dominant pressure frequency at all monitoring positions. At position P2 of the new IGV model, a high amplitude peak takes place at 0.2 BPF, owing to the unsteady turbulent flow developing around the outlet, mid-span of the DV, creating an additional frequency of oscillation, which results in severe vibration and noise. With the new DV model, the number of harmonic frequencies decreases along with the significant reduction in the amplitude. At the BPF and the first higher-harmonic frequency, the amplitude of the new IGV and DV model is reduced, on average, by 12.92% and 22.5%, respectively, compared to that of the new IGV model. This shows that using the new DV brings a positive effect to the sound and vibration. Moreover, the number of small-amplitude peaks at neighboring BPF is also reduced, indicating that the small fluctuations in the DV passage are eliminated in the new DV model, resulting in smooth and stable flow in the DV passage.

23 $${\text{BPF }} = \frac{{N \times {\text{rpm}}}}{60}$$

**Figure 17:**
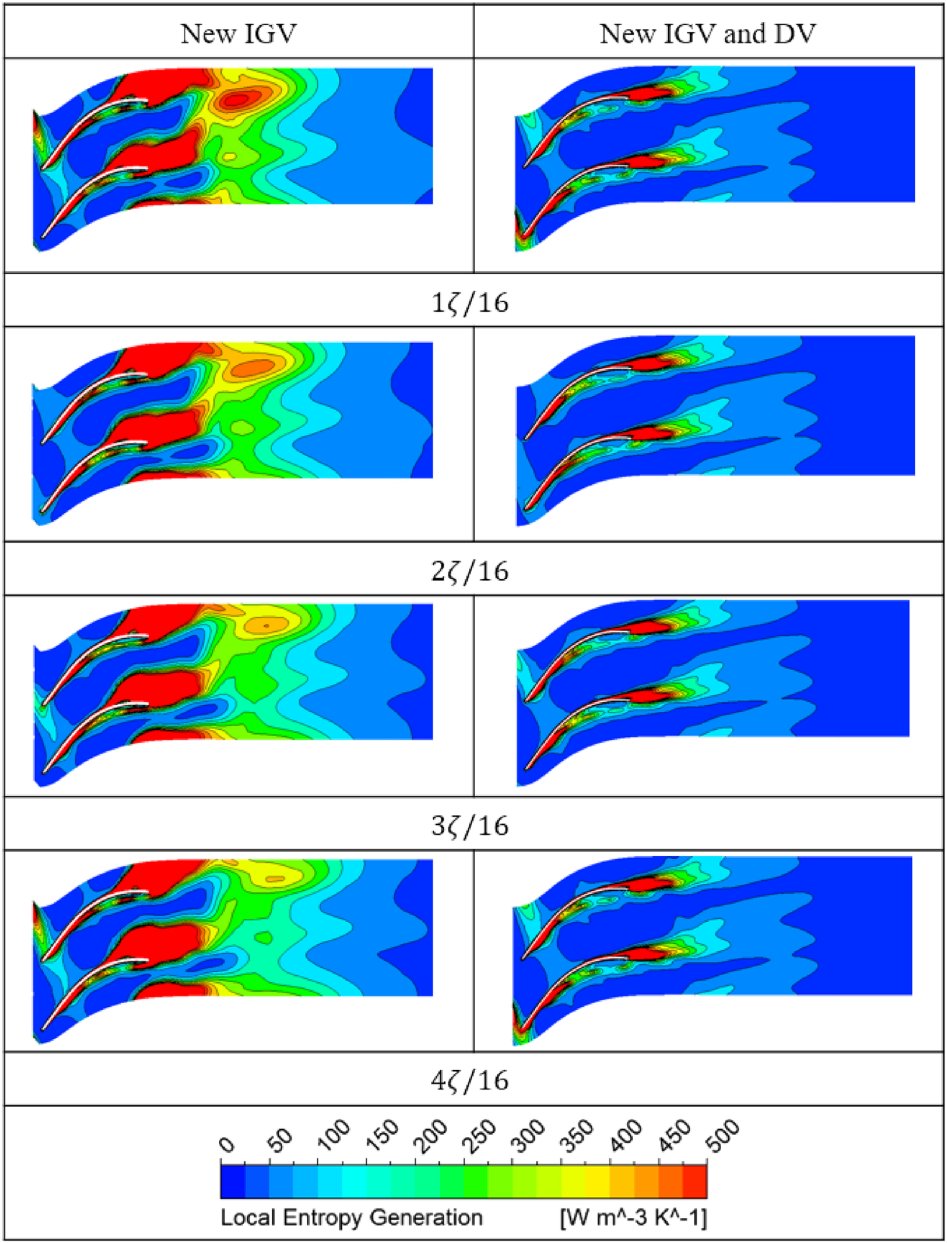
Unsteady distribution of the local entropy generation during a quarter revolution of the impeller at the BEP.

The Fig. 17 shows the evolution of the unsteady entropy generation at 50% span of the DV between the new IGV model and the new IGV and DV model. The local entropy generation contours are compared within a quarter rotation of the impeller ($\zeta$), which is divided into four time steps to reveal the alteration in the internal flow physics of the DV passage. With the new IGV and DV model, the region of high entropy generation is clearly smaller than that in the new IGV model. Moreover, the oscillations in these high-entropy regions are almost nonexistent. There is only a slight alteration in the high-entropy region at the LE of the DV. However, the original state is resumed at the end of a quarter rotation ($4\zeta /16$). This reveals the stability in the fluid flow inside the DV passage of the new DV model. Thereby, the amplitude at the BPF and the harmonic frequencies in the FFT analysis are reduced, leading to a reduction in the vibration and noise. For the new IGV model, the amount of entropy generated around the TE region of the DV is quite high, owing to the flow separation. Furthermore, the fluctuation in the flow is also high because of the formation and growth of a high-entropy region on the TE distal side of the DV. This high-entropy region grows significantly at $1\zeta /16$ and then decreases as the impeller completes a quarter rotation. In addition, this oscillation is non-periodic, as it does not return to its original state at $4\zeta /16$. This indicates the complexity of the flow in the new IGV model, which leads to high amplitudes at the BPF and many other small amplitudes at the harmonic frequencies in the FFT analysis. Consequently, instability develops, causing noise and vibration.

### Energy performance of adjustable IGV and DV

#### Positive DV setting angle

**Figure 18:**
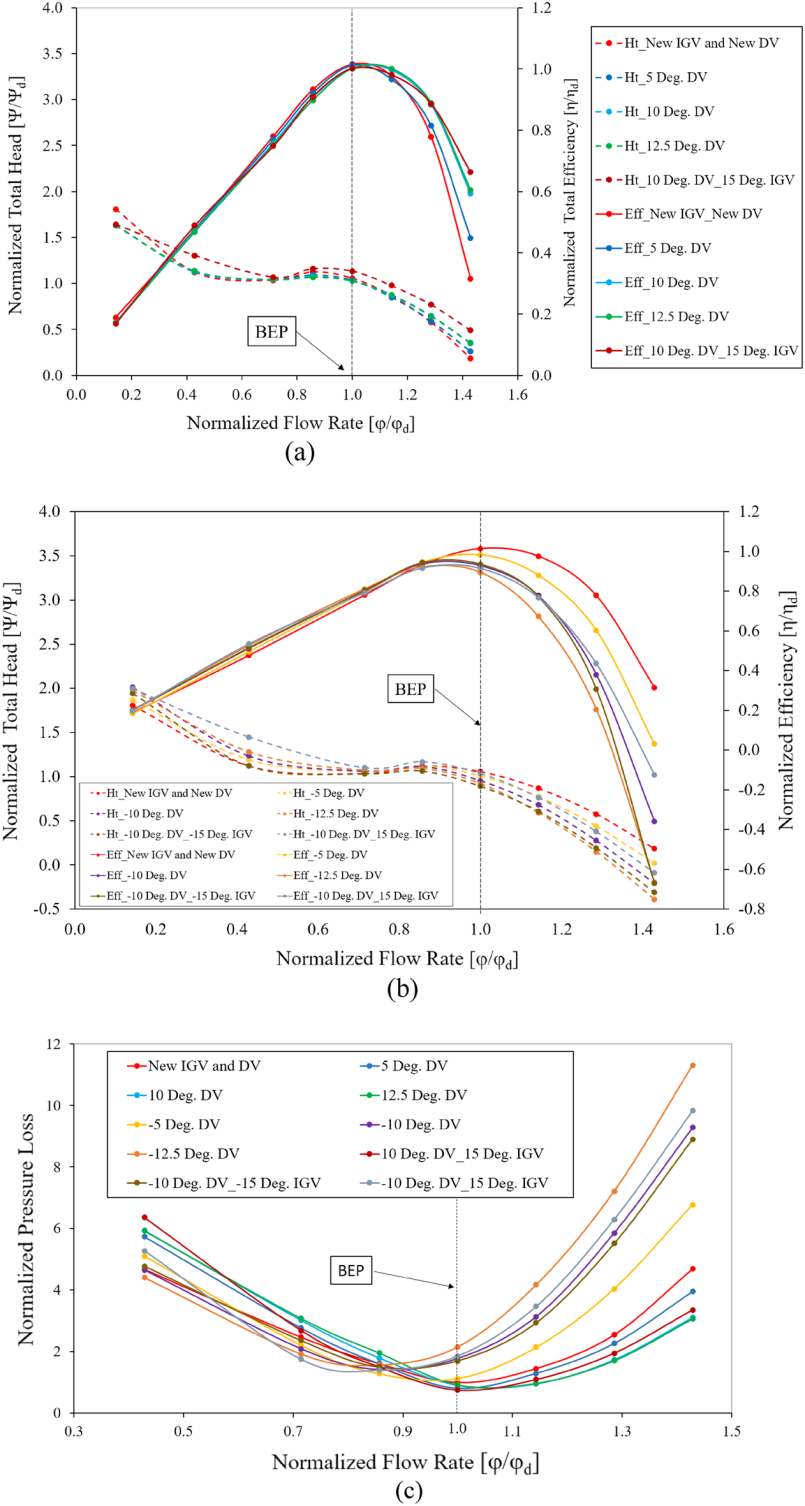

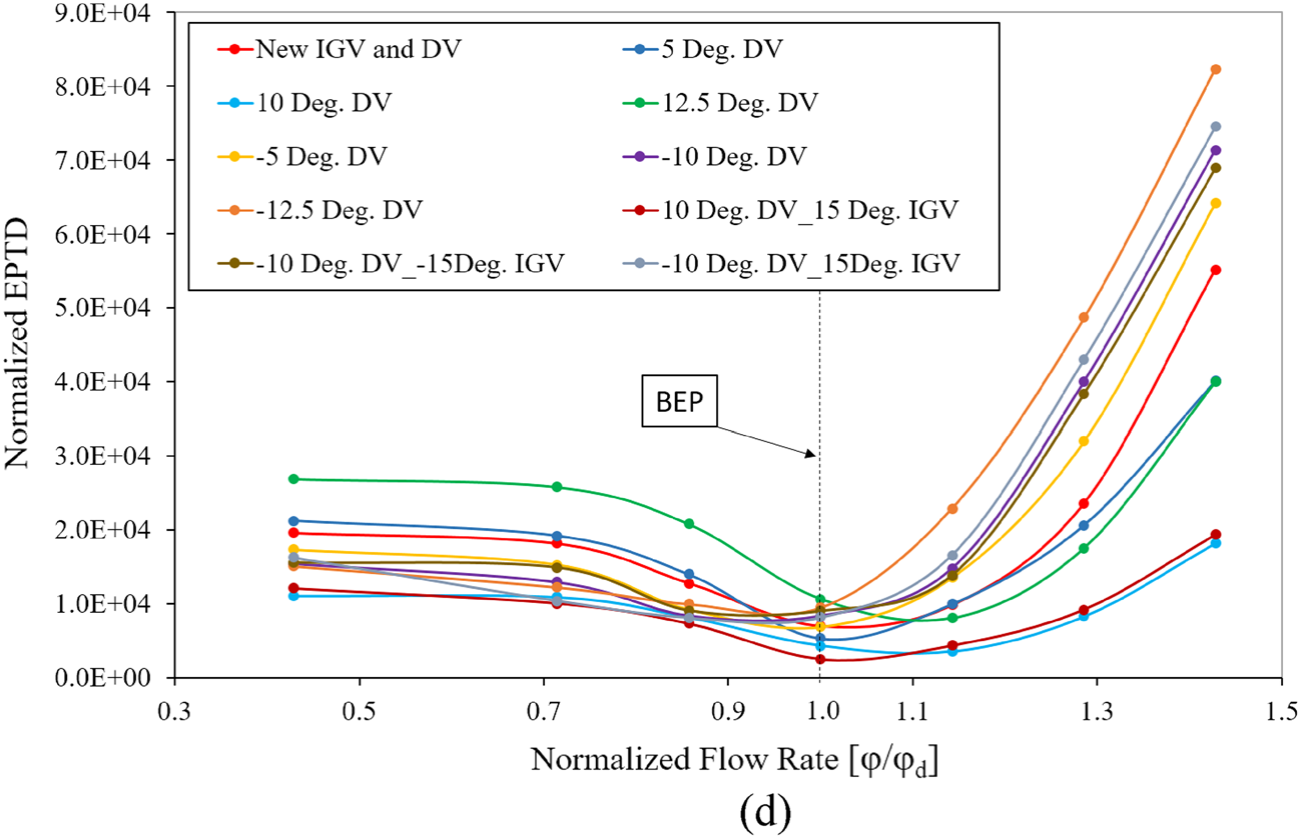
Energy characteristic curves. (**a**) Energy performance of positive DV setting angles, (**b**) Energy performance of negative DV setting angles, (**c**) Pressure loss, (**d**) EPTD.

To enhance the energy performance at the off-design points of the axial-flow pump, an adjustable DV is proposed, with detailed analysis in this section. The new IGV and DV model is used as the base model to investigate the difference in the energy performance of various DV setting angles. Figure 18a shows the energy performance of positive DV setting angles under different flow rates. The efficiency and total head values are normalized using their values under the design conditions of the reference model. It is evident that the energy performance in the low-flow-rate region is adversely affected by the positive setting angle of the DV, owing to the mismatching of the flow and blade angle at the LE of the DV, causing separation flow as shown in Fig. 2. In contrast, the efficiency rises significantly in the high-flow-rate region as the rotating angle increases because of the compatibility of flow and DV blade angles, particularly at a flow rate of 1.43 ${\varphi }_{d}$. The energy performance at the BEP does not improve, as the blade angle of the reference DV is initially optimized to obtain the highest performance at the BEP. Although the efficiency is high in the high-flow-rate region, the total head increase is quite small, indicating that the increase in efficiency is due to the reduction of the energy dissipation in the fluid flow. When the DV setting angle reaches $12.5^\circ$, the efficiency and head are almost unchanged from that at $10^\circ$. Consequently, $12.5^\circ$ is considered as the critical setting angle of the DV. At a flow rate of 1.29 ${\varphi }_{d}$, the efficiency at DV setting angles of $5^\circ$, $10^\circ$, and $12.5^\circ$ increases by 4.64%, 13.52%, and 14.29%, respectively, compared to the efficiency exhibited by the new IGV and DV model. Meanwhile, the total head increases by 2.47%, 11.67%, and 12.19%, respectively. As shown in a previous study^6^, incorporating an adjustable IGV further increases the total head, producing improved energy performance. The adjustable IGV mainly increases the total head, whereas the adjustable DV increases the efficiency. Combining the two, the efficiency of the $10^\circ$ DV and $15^\circ$ IGV setting angle model at a flow rate of 1.29 ${\varphi }_{d}$ is virtually unchanged, whereas at 1.43 ${\varphi }_{d}$, it increases slightly over the model of $10^\circ$ DV setting angle. Meanwhile, the total head increases up to 19.48% and 41.07% at flow rates of 1.29 ${\varphi }_{d}$ and 1.43 ${\varphi }_{d}$, respectively. This is extremely beneficial to the application of an axial-flow pump, in which, at the same head, the pump can operate with a larger flow rate while maintaining the same efficiency.

The Fig. 18c presents the hydraulic loss in the DV passage at different setting angles under various flow rates. The hydraulic loss values are non-dimensionalized by its value under the design conditions of the reference model. The hydraulic loss at a flow rate of 0.143 ${\varphi }_{d}$ is too large to be included in the chart. As mentioned above, the efficiency of the axial-flow pump is closely related to the hydraulic loss. In the high-flow-rate region, the hydraulic losses of the $10^\circ$ and $12.5^\circ$ setting angle models are the smallest, corresponding to the highest efficiency. In the low-flow-rate region of the positive setting angles, the hydraulic loss is much higher than that of the new IGV and DV model, reducing the efficiency. At 1.43 ${\varphi }_{d}$, the hydraulic loss of the $10^\circ$ DV and $15^\circ$ IGV setting angle model is higher than that of the $10^\circ$ DV and $12.5^\circ$ DV setting angle models, whereas the efficiency is greater. This is explained by the high increase in the flow energy transferred by the rotating impeller, leading to a sharp increase in the total head and resulting in increased efficiency. The rotation of the IGV changes the fluid flow characteristics, causing the hydraulic loss in the DV to increase accordingly. Figure 18d depicts the local entropy generation induced by the turbulence dissipation in the DV passage at various setting angles under different flow rate conditions. In the high-flow-rate region, the EPTD seems to correspond with the hydraulic loss with the enhancement of entropy generation. The high entropy generation in the $12.5^\circ$ DV setting angle model causes its efficiency no longer increase compared to that of $10^\circ$. Therefore, $12.5^\circ$ is considered as the critical setting angle of the DV, as mentioned above. The EPTD does not tend to rise with decreasing flow rate, because the velocity in the DV passage is smaller under a low-flow-rate, reducing the rate of energy dissipation. This results in less entropy generation than that under high-flow-rate conditions.

**Figure 19:**
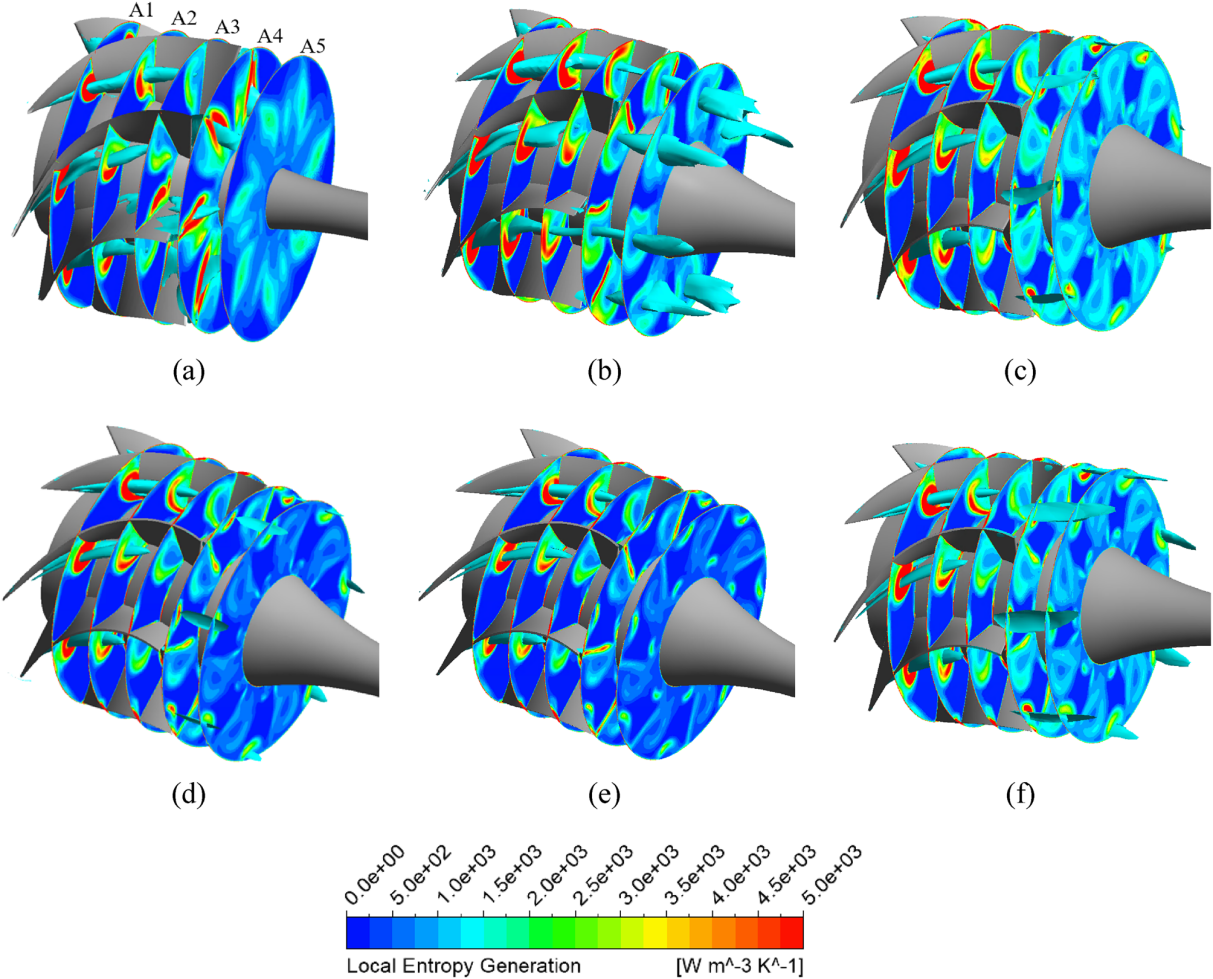
Distribution of the local entropy generation and low-velocity zone in the DV passage under 1.29 ${\varphi }_{d}$. (**a**) Reference, (**b**) New IGV and DV, (**c**) $5^\circ$ DV, (**d**) $10^\circ$ DV, (**e**) $12.5^\circ$ DV, (**f**) $10^\circ$ DV and $15^\circ$ IGV.

The Fig. 19 shows the local entropy generation in multiple cross-sections in the DV passage for the reference model, new IGV and DV model, and different positive DV setting angle models under a flow rate of 1.29 ${\varphi }_{d}$. The three-dimensional distributions of the low-velocity zone in the DV passage with a velocity coefficient of 0.08 are also presented in cyan. Evidently, the low-velocity zone distributed inside the DV passage is associated with the region of high entropy generation, considered as the core of the high-entropy region. Furthermore, the high-entropy generation region follows the trajectory of the low-velocity zone. This demonstrates that the turbulent flow swirls around the core of the low-velocity region, causing large energy dissipation. Additionally, it also indicates that low-velocity zones under high-flow-rate conditions generate a significant amount of turbulent energy, which causes energy dissipation and increases in both hydraulic loss and entropy generation. Under a flow rate of 1.29 ${\varphi }_{d}$, the low-velocity zones are formed by flow separation at the PS of the DV, starting at LE and extending inside the DV passage. As can be seen, the low-velocity zone and high-entropy region of the new DV model are slightly larger than those in the reference model because of the further growth of the low-velocity core across plane A5, as shown in Fig. 19b. However, this does not cause too much entropy generation because of its stability. This is why the efficiency of the new DV model is only slightly lower than that of the reference model. This greatly improves with DV rotation, especially at the DV setting angles of $10^\circ$ and $12.5^\circ$. The high-entropy and low-velocity regions of these models are clearly reduced compared to both the new DV and reference models. With the combination of the DV rotating simultaneously with the IGV adjusted to $15^\circ$, the entropy intensity is only slightly larger than that of the $10^\circ$ DV setting angle model, but it is still less than the $5^\circ$ DV model. Meanwhile, the low-velocity region inside the DV passage remains almost unchanged; there is only an additional low-velocity region at the shroud behind the TE, as shown in Fig. 19f. This is due to the change in the fluid flow characteristics throughout the pump owing to IGV rotation. This low-velocity region leads to a slightly higher hydraulic loss than that of the $10^\circ$ DV setting angle model.

**Figure 20:**
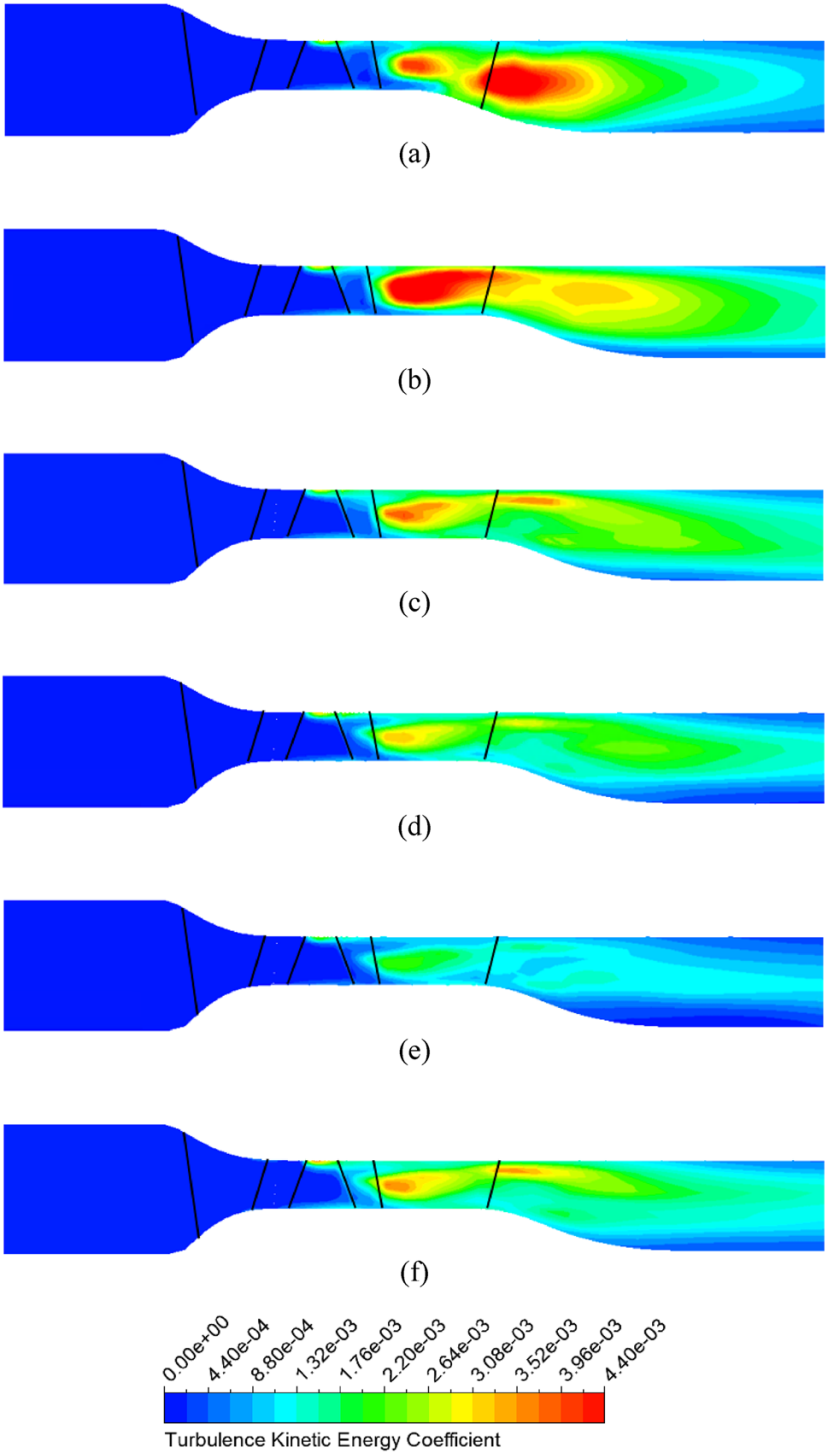
TKE in the meridional plane under 1.29 ${\varphi }_{d}$. (**a**) Reference, (**b**) New IGV and DV, (**c**) $5^\circ$ DV, (**d**) $10^\circ$ DV, (**e**) $12.5^\circ$ DV, (**f**) $10^\circ$ DV and $15^\circ$ IGV.

Pertaining to the location of the position of energy loss, Fig. 20 depicts the TKE at the meridional plane for the reference model, new IGV and DV model, and different positive DV setting angle models under the flow rate of 1.29 ${\varphi }_{d}$. For the new DV model, the turbulence behind the TE of the DV is significantly improved with a decrease in the TKE. However, a strong turbulent flow develops inside the DV passage, leading to high-energy loss and performance degradation. With DV rotation, the TKE is gradually reduced with an increase in the setting angle. The turbulence originating from the LE of the DV is improved by the compatibility of the flow and blade angles, leading to a reduction in turbulence energy and increase in efficiency. When combined with the IGV adjusted to $15^\circ$, the turbulence intensity increases slightly compared to that of the model with a DV setting angle of $10^\circ$, as shown in Fig. 20f. The low-velocity zone in the shroud region behind the TE of the DV clearly produces significant TKE, which does not increase the performance of the pump in comparison with that of the $10^\circ$ DV setting angle model.

#### Negative DV setting angle

The Fig. 18b presents the energy performance of the negative DV setting angles under different flow rates. The efficiency and total head are normalized based on their values under the design conditions of the reference model. In contrast to the positive setting angles, the negative setting angles produce an energy performance in the high-flow-rate region that is significantly reduced compared to that of the new IGV and DV model. This is due to the occurrence of flow separation in the PS of the DV, caused by the mismatching between the flow and blade angles, as can be seen in Fig. 2. However, the energy performance of the negative setting angles is slightly enhanced in the low-flow-rate region, especially under the flow rates of 0.43 ${\varphi }_{d}$ and 0.71 ${\varphi }_{d}$. Specifically, at the $-10^\circ$ setting angle of the DV model, the efficiency is greater than that of the new IGV and DV model, by up to 7.33% and 2.77% under the flow rates of 0.43 ${\varphi }_{d}$ and 0.71 ${\varphi }_{d}$_,_ respectively. Likewise, at the $-12.5^\circ$ setting angle of the DV model, the efficiency is greater by 10.52% and 3.96%, respectively. The total head moderately increases at 0.71 ${\varphi }_{d}$ while increasing sharply at 0.43 ${\varphi }_{d}$, with the highest increase of up to 14.27% corresponding to the $-12.5^\circ$ model. This enhancement in energy performance shows that the separation flow in the SS of the DV under low flow rates is significantly improved. To further increase the total head at a low-flow-rate conditions, two IGV setting angle models are proposed to simultaneously combine with the $-10^\circ$ DV setting angle model. The results show that, for the $-15^\circ$ IGV setting angle model incorporated with the DV, the total head does not seem to increase compared to that of the new IGV and DV model. However, for the $15^\circ$ IGV incorporated with the DV, the total head pressure rises slightly at 0.71 ${\varphi }_{d}$ and sharply at 0.43 ${\varphi }_{d}$, by 6.41% and 29.03%, respectively. Along with the increase in the total head, the efficiency at 0.43 ${\varphi }_{d}$ also increases significantly by 11.96%. Based on the above analysis, it can be stated that the positive setting angle of IGV has a positive effect on the total head, and the negative setting angle of DV can surely increase the energy performance of the axial-flow pump under low-flow-rate conditions.

Considering the close relationship between hydraulic loss and efficiency, the efficiency for the negative DV setting angle models is smaller than that of the new IGV and DV model in the high-flow-rate region owing to the large hydraulic loss, as shown in Fig. 18c. However, this negative effect is notably improved in the low-flow-rate region, leading to a slight increase in the energy performance. This reduction in hydraulic loss indicates that the turbulent areas in the DV passage are significantly improved. Although combination with the $15^\circ$ IGV setting angle model brings higher hydraulic losses, energy performance is still at a higher level than that of the new IGV and DV model. This is due to how much more energy is transferred to the fluid flow by the rotating impeller than by the actual hydraulic loss. The rotating impeller increases the total head and, thereby, increases the efficiency of the axial-flow pump. As with the tendency of the positive DV setting angle, shown in Fig. 18d, the EPTD trend of the negative DV setting angle is also consistent with that of the hydraulic loss, corresponding to an increase in entropy generation under high-flow-rate conditions owing to large turbulence. The entropy generation in the low-flow-rate region is smaller than that of the new IGV and DV model, resulting in increased efficiency.

**Figure 21:**
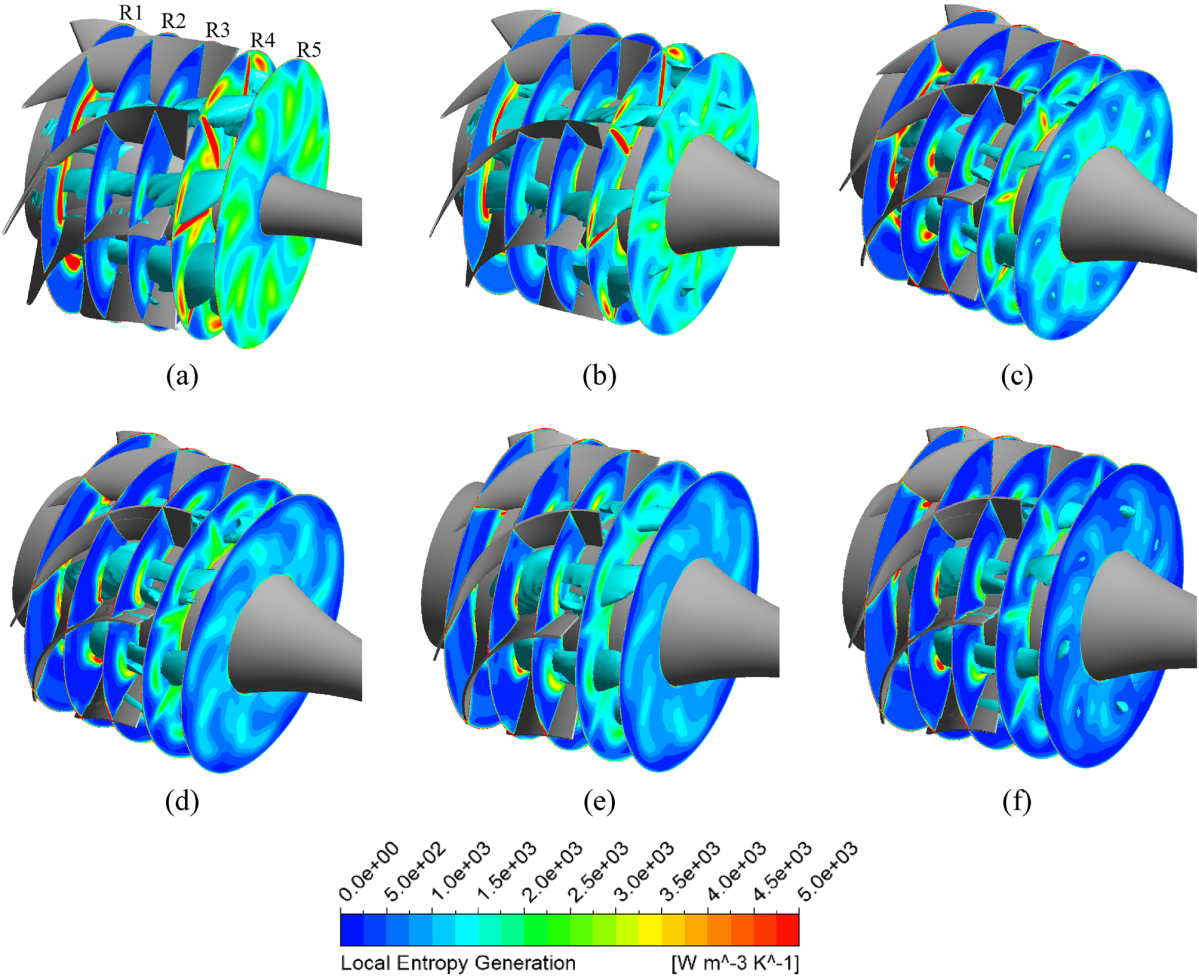
Distribution of the local entropy generation and low-velocity zone in the DV passage under 0.71 ${\varphi }_{d}$. (**a**) Reference, (**b**) New IGV and DV, (**c**) $-5^\circ$ DV, (**d**) $-10^\circ$ DV, (**e**) $-12.5^\circ$ DV, (**f**) $-10^\circ$ DV and $15^\circ$ IGV.

The Fig. 21 depicts the local entropy generation among the different negative DV setting angle models in various planes in the DV passage under a flow rate of 0.71 ${\varphi }_{d}$. The development of low-velocity zones in the DV passage with a velocity coefficient of 0.04 is also shown in cyan. In contrast to the BEP and high-flow-rate regions, the low-velocity zone under low flow rates in the DV passage is not completely associated with the region of high entropy generation. In fact, the low-velocity zone even passes through low-entropy regions. The high-entropy regions are distributed mainly around the vicinity of the low-velocity zone. This indicates that the velocity gradient in the low-velocity zone is low, which thereby reduces the rate of entropy dissipation. This is reasonable because a decrease in the flow rate results in a reduction in the velocity of the fluid flow. Therefore, the energy loss owing to entropy generation under low-flow-rate conditions is lower than that under high-flow-rate conditions. The entropy generation at plane R1 is quite high near the hub, owing to the large kinetic energy of the flow coming from the rotating impeller. Additionally, because of the presence and extensive propagation in the DV passage of these low-velocity zones starting from R1, the hydraulic loss is significantly increased in the low-flow-rate region. These low-velocity zones are gradually improved over the different negative setting angles of the DV. With the DV rotations of $-10^\circ$ and $-12.5^\circ$, the low-velocity zones do not appear on plane R5. The local entropy generation is also clearly reduced at these setting angles. The high-entropy regions on plane R1 are almost eliminated. Owing to the low energy performance, further analysis of the $-10^\circ$ DV setting angle combined with the $-15^\circ$ IGV setting angle is omitted. With the combination including the $15^\circ$ setting angle of the IGV, the entropy generation and low-velocity zones are slightly diminished in comparison with those of the $-10^\circ$ DV model, as shown in Fig. 21f. This is because the change in the IGV setting angle leads to a change in the fluid flow physics inside the axial-flow pump. The flow leaves the impeller with high energy and velocity which reduces the flow angle at the LE of the DV relative to the rotating axis, resulting in less turbulent flow and entropy generation in the SS of the DV.

**Figure 22:**
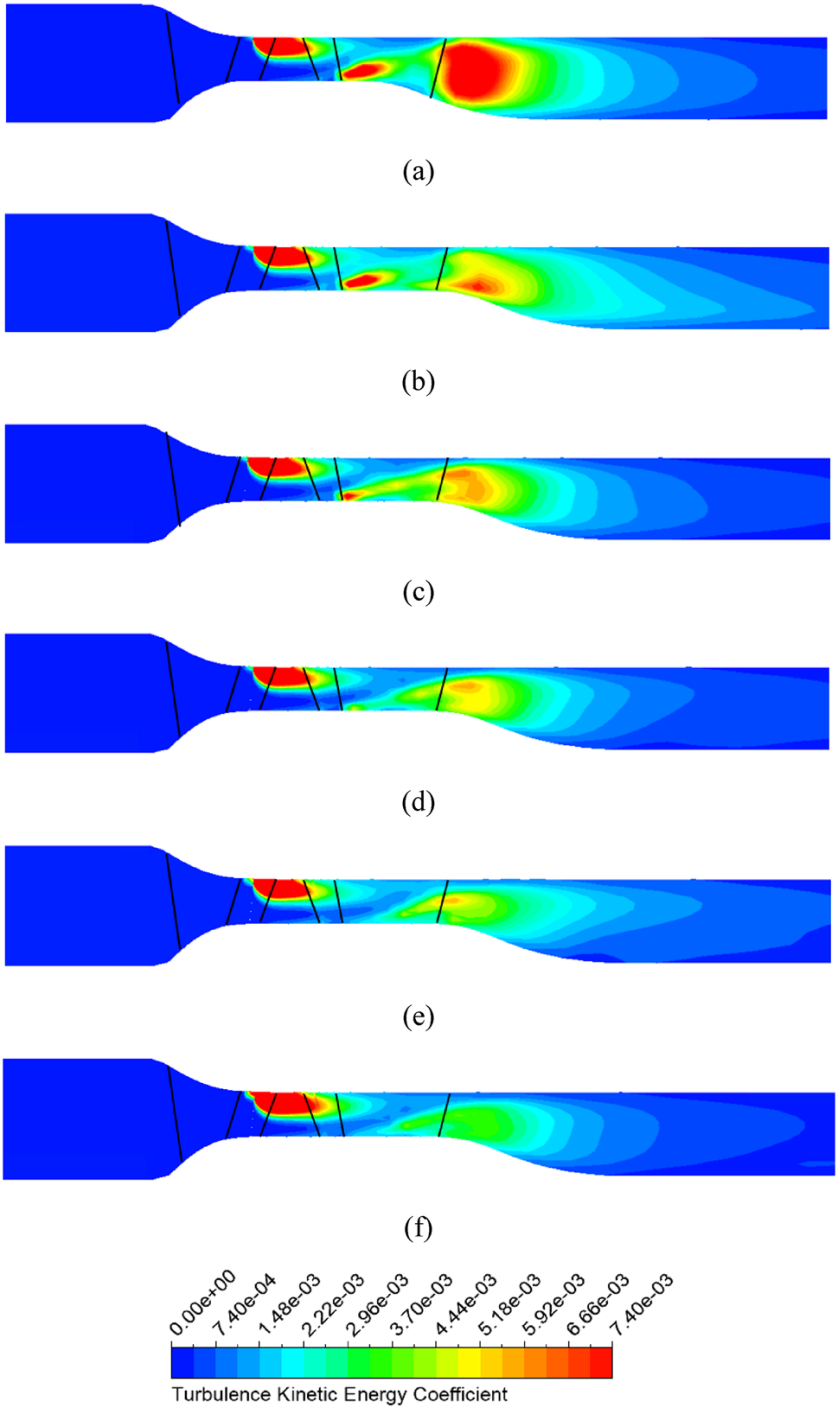
TKE in the meridional plane under 0.71 ${\varphi }_{d}$. (**a**) Reference, (**b**) New IGV and DV, (**c**) $-5^\circ$ DV, (**d**) $-10^\circ$ DV, (**e**) $-12.5^\circ$ DV, (**f**) $-10^\circ$ DV and $15^\circ$ IGV.

The Fig. 22 presents the TKE at the meridional plane for the reference model, new IGV and DV model, and various negative DV setting angle models under the flow rate of 0.71 ${\varphi }_{d}$. With the new DV, the TKE is considerably improved by the reduction of the turbulent flow behind the TE of the DV. Additionally, the turbulent flow inside the DV passage near the LE is also slightly decreased. The turbulent flow in the DV passage decreases under low flow rates and around the BEP, whereas it increases under high-flow-rate conditions. This indicates that the cross-sectional area of the DV has a great influence on the amount of turbulent flow inside the DV passage. The above analysis shows that under any flow rate condition, the amount of turbulent flow behind the TE of the DV is also notably improved compared to that of the reference DV model. With a decrease in the DV setting angle, the TKE is gradually reduced. In particular, the turbulent flow originating from the LE of the DV is completely suppressed for the $-12.5^\circ$ DV setting angle of the DV because of the reduction in the TKE. Moreover, the decrease in TKE behind the TE of the DV also suggests that the turbulent flow around this area is also significantly improved. The TKE is smallest when the DV is simultaneously combined with the $15^\circ$ IGV model, as can be seen in Fig. 22f. The amount of TKE behind the TE of the DV is clearly less than that exhibited in the DV model with $-10^\circ$ setting angle. Consequently, operating with positive setting angles of the IGV also brings a positive effect on the energy performance of the axial-flow pump under low-flow-rate conditions.

## Conclusion

This study analyzes the effects of the IGV and DV hub profiles of the axial-flow pump on energy performance. The hydrofoil profile from the NACA foil is employed in the IGV geometry, and the DV hub is designed with a straight profile. In addition, an adjustable DV is thoroughly investigated through entropy generation theory to evaluate its enhancement of the energy performance of the axial-flow pump at the off-design points. Furthermore, combinations of the simultaneously adjustable IGV and adjustable DV are also performed and analyzed in detail. Some key conclusions are drawn as follows.

1. The new IGV with a hydrofoil profile has a positive effect on the energy performance of the axial-flow pump, with a 3.75% increase in the total head at the BEP, compared to that of the reference model. This increase is obtained by reducing the absolute flow angle at the LE of the impeller, which facilitates the fluid flow into the impeller with higher energy. Although the flow velocity increases, the local entropy generation in the rotating impeller is negligible. The high entropy generated at the tip clearance and behind the TE of the impeller by the TLV and flow separation, respectively, is significant but inevitable.
2. The cross-sectional area inside the DV has a significant influence on the formation and development of the turbulent flow in the DV passage. Operating with new IGV and DV models, the efficiency and total pressure head are greater than those of the reference model at the BEP, by 1.456% and 5.756%, respectively. This is mainly due to the improvement in the turbulent flow behind the TE of the DV. This improvement also results in a decrease in the entropy generation in the DV passage, compared to that of the new IGV model, with a 43.38% reduction in EPTD. Additionally, the entropy generation caused by turbulence dissipation is a major component of the energy loss that takes place in the DV passage compared to one caused by direct dissipation. Using the new DV also reduces the vibration and noise caused by the unsteady turbulent flow in the DV passage.
3. Positive DV setting angles increase the energy performance of the axial-flow pump under high-flow-rate conditions. The $10^\circ$ is considered the critical positive setting angle of the DV, with efficiency and total head greater than those of the new IGV and DV model, by 13.52% and 11.67%, respectively. With the combination of $15^\circ$ IGV and $10^\circ$ DV model, the total head is greater than that of the $10^\circ$ DV model, by 19.48% and 41.07% under flow rates of 1.29 ${\varphi }_{d}$ and 1.43 ${\varphi }_{d}$, respectively. The low-velocity zones, entropy generation, and hydraulic losses are all significantly improved in the high-flow-rate region. In addition, the low-velocity zone is also found to be closely related to the high-entropy region at the BEP and high-flow-rate conditions, as the high-entropy regions follow the trajectory of the low-velocity zones.
4. The energy performance in the low-flow-rate region increases for negative DV setting angles, owing to the reduction in hydraulic loss and entropy generation in the DV passage. The efficiency and total head exhibit the greatest increase at $-12.5^\circ$ DV model, by 10.52% and 14.27%, respectively, compared to those of the new IGV and DV model, under a flow rate of 0.43 ${\varphi }_{d}$. In the combined DV model with adjustable IGV, the $-15^\circ$ IGV is shown be less effective, whereas the $15^\circ$ IGV produces a notable increase in efficiency and total head over that of the new IGV and DV model, by 11.96% and 29.03%, respectively, under a flow rate of 0.43 ${\varphi }_{d}$. Therefore, positive IGV setting angles are advantageous to axial-flow pump operation. In the low-flow-rate region, the entropy generation decreases sharply over the high-flow-rate region owing to the decrease in velocity gradient; meanwhile, the hydraulic loss rises sharply because of the expansion of the low-velocity zone. In addition, the low-velocity region is not closely related to the high-entropy region in the DV passage under low-flow-rate conditions. Under any flow rate, the turbulent flow behind the TE of the DV is improved with the operation of the new DV.

## Data Availability

The data that use and analyze in this study are available from the corresponding authors upon reasonable request.
